# The association between parental BMI and offspring adiposity: A genetically informed analysis of trios

**DOI:** 10.1371/journal.pgen.1011775

**Published:** 2025-08-05

**Authors:** Liam Wright, Gemma Shireby, Tim T. Morris, Neil M. Davies, David Bann

**Affiliations:** 1 Centre for Longitudinal Studies, Social Research Institute, University College London, London, United Kingdom; 2 Division of Psychiatry, University College London, London, United Kingdom; 3 Department of Statistical Science, University College London, London, United Kingdom; 4 Department of Public Health and Nursing, Norwegian University of Science and Technology, Trondheim, Norway; Newcastle University, UNITED KINGDOM OF GREAT BRITAIN AND NORTHERN IRELAND

## Abstract

**Background:**

Children with obesity are more likely to have parents with obesity than those without. Several environmental explanations have been proposed for this correlation, including foetal programming and parenting practices. However, body mass index (BMI) is a heritable trait; child-parent correlations may reflect direct inheritance of adiposity-related genes. There is some evidence that mothers’ BMI associates with offspring BMI net of direct genetic inheritance, consistent with both intrauterine and parenting effects, but this requires replication. Here, we also investigate the role of fathers’ BMI as well as offsprings’ diet as a mediating factor.

**Methods:**

We used Mendelian Randomization (MR) with genetic trio (mother-father-offspring) data from 2,630 families in the Millennium Cohort Study, a UK birth cohort study of individuals born in 2000/02, to examine the association between parental BMI (kg/m^2^) and offspring birthweight and BMI and diet measured at six-time points between ages 3y and 17y. Paternal and maternal BMI were instrumented with polygenic indices (PGI) for BMI conditioning upon offspring PGI. This allowed us to separate direct and indirect (“genetic nurture”) genetic effects. We compared these results with associations obtained using standard multivariable regression techniques using phenotypic BMI data only.

**Results:**

Mothers’ and fathers’ BMI were positively associated with offspring BMI to similar degrees. However, in MR analysis, associations between father’s BMI and offspring BMI were close to the null. In contrast, mother’s BMI was consistent in MR analysis with phenotypic associations. Maternal indirect genetic effects were between 25–50% the size of direct genetic effects. There was limited and inconsistent evidence of associations with offspring diet and some evidence that mothers’, but not fathers’, BMI was related to birthweight in both MR and multivariable regression models.

**Conclusions:**

Results suggest maternal BMI may be particularly important for offspring BMI: associations may arise due to both direct transmission of genetic effects and indirect (genetic nurture) effects. Associations of father’s and offspring adiposity that do not account for direct genetic inheritance may yield biased estimates of paternal influence. Larger studies are required to confirm these findings.

## Author summary

Children with obesity are more likely to have parents with obesity, too. This may be explained by environmental factors, such as prenatal conditions in utero or postnatal parental behaviours (e.g., the foods parents provide). If this is the case, interventions to reduce parental body mass index (BMI) could have intergenerational effects. However, the correlation between parent-child adiposity may reflect children inheriting obesity-related genes from their parents. To explore this, we used genetic data from over 2,500 trios (mother-father-offspring sets) to assess whether parental obesity-related genes are associated with offspring adiposity and diet after accounting for direct genetic inheritance. We compared this to analyses that do not account for genetic inheritance. We found that, though mothers’ and fathers’ BMI were consistently correlated with children’s BMI, after accounting for direct genetic inheritance, this only remained true for mothers during the child’s adolescence. Associations of parents’ BMI and children’s diet were less consistent. Our results suggest that associations between fathers’ and children’s adiposity that do not account for direct genetic inheritance yield biased estimates of paternal influence.

## Introduction

Obesity rates among children and adolescents have risen sharply over the past five decades [1]. This has motivated a large body of research on factors that contribute to high body mass index (BMI) among young people [2,3]. One focus of this research has been on the role of parents, particularly the role of parental adiposity. Children with obesity are more likely to have parents with obesity, too [4–7].

Several reasons have been given to explain this correlation. The developmental overnutrition hypothesis [DOH; 8] posits that higher levels of maternal adiposity while the child is *in utero* can have long-term effects on offspring BMI by increasing circulating levels of pro-inflammatory cytokines, glucose, and fatty acids in the mother [9–11]. These are thought to increase birth weight and ‘program’ permanent changes to offspring adiposity-related physiology and behavioural traits, including changes to appetite control and metabolism [9–13].

However, the DOH cannot explain the correlation between father’s and offspring BMI. Factors upstream or downstream of father’s (and mother’s) adiposity, such as diet and exercise behaviour, could instead be important postnatally through their influence on the postnatal environment in which the child develops. Aspects of the family and home environment that are particularly pertinent to BMI are parental diet (which influences food availability and behaviour modelling), *food parenting practices* (active behaviours and influences on children’s diet) [14], and analogous influences on exercise and physical activity [15,16].

While these processes may explain a correlation between parental and offspring adiposity, BMI is a heritable – and polygenic [17,18] – trait; heritability estimates from twin studies range 47–90% [19,20], while an estimate using identity-by-descent in siblings was 42% [21]. Therefore, the association between parents and their children may reflect direct transmission of genes passed on to offspring rather than the influence of parental traits and behaviours.

**Fig 1:**
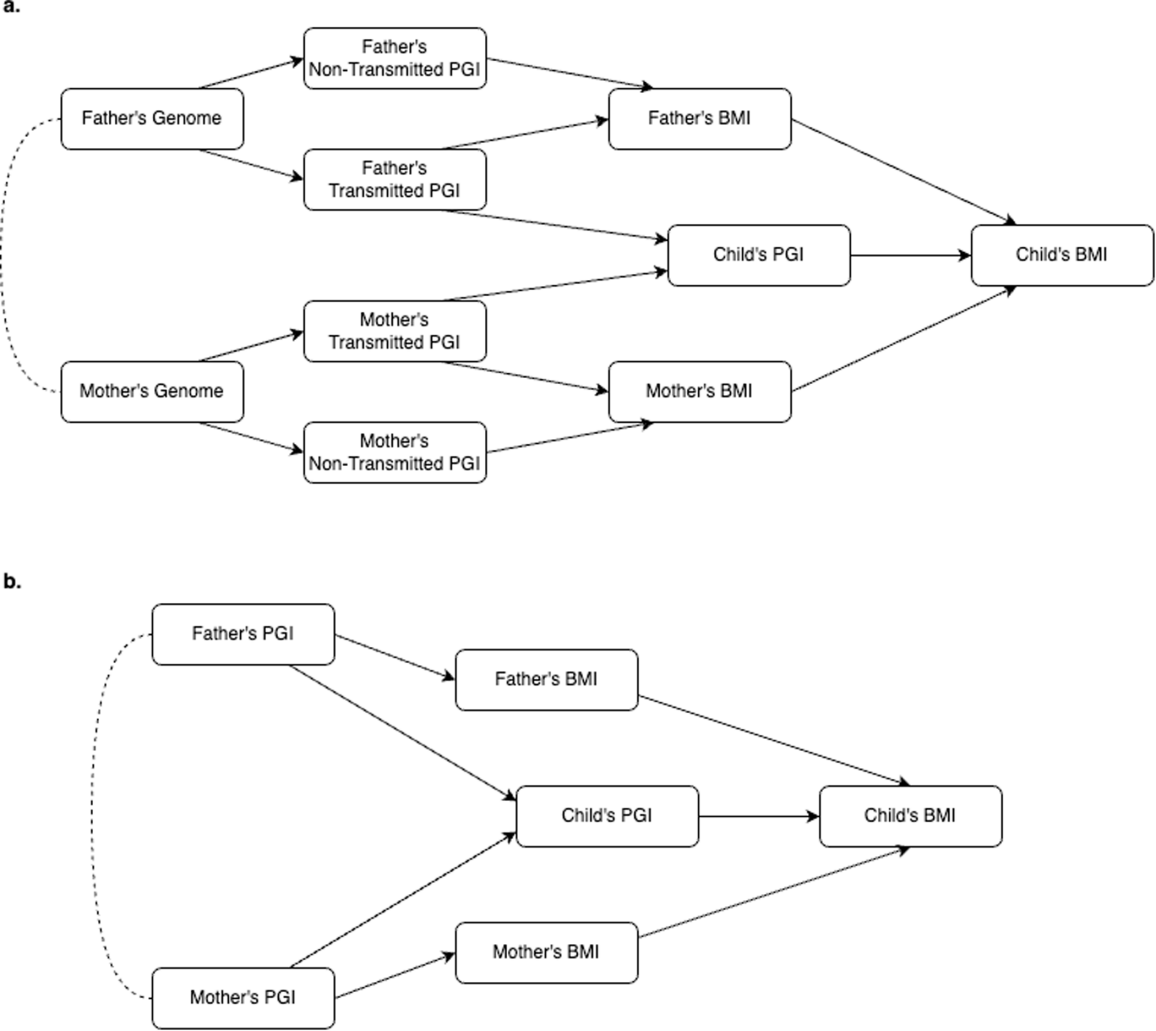
Directed Acyclic Diagram of the relationship between parental PGIs and child’s BMI. Mother’s and father’s PGI are hypothesised to be causally related to child’s BMI via transmission of genetic material to the child (i.e., through inerited alleles, as captured in the child’s PGI) and indirectly via non-transmissed alleles that influence parental BMI and consequent traits and behaviours (intrauterine environment, food parenting practices, etc.). In **(a)**, mother’s and father’s genomes are split into transmitted and non-transmitted alleles before PGIs are calculated. In this DAG, mother’s non-transmitted PGI is a valid instrument for mother’s BMI, conditional upon the father’s transmitted and non-transmitted PGIs. Without conditioning, estimates would be biased due to assortative mating (dashed line). It is not necessary to condition upon child’s PGI to obtain unbiased estimates. In **(b)**, PGIs are calculated using all alleles. Mother’s PGI is a valid instrument for mother’s BMI, conditional upon child’s PGI and father’s PGI. If father’s PGI is not controlled for, mother’s PGI is an invalid instrument for two reasons: first, confounding due to assortative mating (dashed line); second, collider bias as adjusting for the child’s PGI opens a path between father’s PGI and mother’s PGI. In the DAG, mother’s PGI would also be a valid instrument if father’s BMI was controlled for. However, in practice, father’s BMI is likely to be measured with error (e.g., self-report at a single time-point), and thus not would fully close the paths from mother’s PGI to child’s BMI via father’s PGI. Note, if father’s PGI does not have an indirect effect, no bias will arise. An assumption in the DAGs is that parent’s PGI do not influence parental traits and behaviours that themselves influence child’s BMI (in instrumental variable analysis, this is known as the exclusion restriction assumption). For reasons given in the main text, this assumption is likely to be violated in practice. However, the designs shown in (a) and (b) can still account for direct genetic transmission.

Only a few studies have used genetically-informed designs that can account for the direct genetic transmission of adiposity-related genes [16,22–27]. These exploit the fact that children inherit only half of their parents’ genomes and either use polygenic indices (PGI) for BMI constructed using parental ‘non-transmitted’ alleles or adjust for parental and offspring PGIs simultaneously, thus enabling the assessment of ‘genetic nurture’ effects operating indirectly via parental traits and behaviours (Fig 1) [28]. These studies have generated inconsistent results but have differed according to the age of BMI assessment, the predictive power of PGIs used, and whether maternal and paternal genetic effects have been separated. Analyses of one regional UK birth cohort show some evidence of an association between a mother’s BMI PGI and offspring’s BMI in adolescence (22,27, though also see 16,25). Paternal effects were not assessed, however. Studies from Norway [24], Denmark [26] and Iceland [23] found little evidence of association with parental genetics (after accounting for direct genetic transmission) in adolescence, at age 18y or during adulthood, respectively – though, these studies either used a relatively small sample size or did not separate maternal and paternal effects.

The latter three studies are also consistent with a genome-wide association study (GWAS) comparing within- and between-sibling genetic associations that found little evidence of genetic nurture influencing adult BMI [29]. Adoption studies additionally show evidence of weak correlations between children and their adoptive parents’ BMI [30]. However, these studies were carried out before, or in the early stages of, the obesity epidemic.

The current evidence is, therefore, restricted by the partial use of genetic trio (mother-father-offspring) data, a lack of direct examination of paternal effects, and a small number of samples used. Further, there has been no investigation of the pathways through which indirect genetic effects may arise, for instance, effects upon diet, which (as noted) could explain correlations in father-child BMI. Therefore, in this study, we investigated whether maternal and paternal genetics have indirect effects on offspring outcomes by performing an MR analysis of over 2,600 genetic trios in the Millennium Cohort Study, a UK birth cohort with repeat assessment of children’s BMI and diet from early childhood to late adolescence.

## Methods

### Ethics statement

Ethical review for each sweep of the Millennium Cohort Study (MCS) was provided by a National Health Service Research Ethics Committee [approval 17/NE/0341; see 31, for more detail]. Verbal consents were taken from children and written consents from their parents or caregivers.

### Participants

Participants in the MCS were born between 2000/02, decades after childhood obesity rates started to rise in the UK [32,33]. Participants have been followed up on multiple occasions, beginning at 9 months and with the most recent data collection available at age 17y. (An additional sample of 4% of participants was recruited at age 3y.) Cohort members’ caregivers and, latterly, the cohort members themselves, were interviewed at each sweep, and various surveys and biomedical data have been collected. At age 14y, cohort members and their resident biological parents provided saliva samples, which were used for genotyping. Further details on the study, including genetic data collection, are available in the cohort profiles [34,35].

The MCS used a clustered stratified sampling design, with oversamples of children living in disadvantaged areas, from ethnic minority backgrounds, or from Scotland, Wales or Northern Ireland. We limited our analysis to singleton births (i.e., non-twins) of (White) European ancestry/ethnicity (n = 15,456; 81.4% of the recruited sample) as the BMI PGI we used was drawn from a GWAS of European ancestry samples [18]. (For genotyped individuals, European ancestry was determined using the GenoPred pipeline [see below]; for non-genotyped individuals, self-reported White European ethnicity was used.) Only parents biologically related to cohort members were eligible for genetic data collection; biologically related mothers or fathers who did not reside with their children were not followed up. As such, sample sizes for trios were limited. 2,630 families had full genetic trio data (44.8% of those observed with both biological parents resident at age 14y, 17% of the total sample of European ancestry), and 5,357 had genetic data from mother-offspring pairs (59.2% of those observed with resident biological mother at age 14y, 34.7% of the total sample with European ancestry).

### Genotyping

Genotyping was done in the Bristol Genetics Labs (Bristol, UK) using Illumina Infinium global screening arrays-24 v1.0. For more details on the collection of samples, DNA extraction methods, and laboratory procedures, see Fitzsimons et al. [35] and Shireby et al [36]. Genotype calling was performed using GenomeStudio (v2.0, Illumina), and quality control was completed using PLINK1.9 and PLINK2.0 [37]. Samples that could no longer be included in the sample (e.g., due to withdrawn consent) were removed before QC. Individuals were excluded if they had > 2% missing data, excess heterozygosity (>3 standard deviation [SD] from the mean), or X chromosome homozygosity discordant with their reported sex (females excluded with an F value > 0.2 and males with an F value < 0.8), so long as these could not be rectified using family relationships inferred using KING.

Before imputation, single nucleotide polymorphisms (SNPs) were excluded if they had high levels of missing data (> 3%), Hardy-Weinberg equilibrium P < 1e-6 (based on a subset of unrelated, European samples), or minor allele frequency (MAF) < 1%. The genetic data were then recoded as VCF files before uploading to the TOPMed Imputation Server, which uses Eagle2 to phase haplotypes and Minimac4 [38] with the TOPMed reference panel. Imputed genotypes were then filtered with PLINK2.0alpha, excluding SNPs with an R^2^ INFO score < 0.8, and recoded as hard calls into binary PLINK format. Proceeding with PLINK1.9, samples with > 2% missing values were removed, and SNPs were excluded if they had > 3% missing values, > 2 alleles or a MAF of < 1%. Duplicate samples were also removed with samples with higher genotyping rates retained. In these steps, European individuals were identified using the GenoPred pipeline which involved (a) merging the MCS genotypes with data from 1000 genomes Phase 3, (b) linkage disequilibrium pruning overlapping SNPs such that no pair of SNPs within 1000 bp had r^2^ > 0.20 and (c) using an elastic net model to identify Europeans versus non-Europeans.

The genetic data used to construct PGIs were highly accurate. Per family, the mean (median) number of SNPs with Mendelian errors was 0.08% (0.09%), and the family with the maximum number of Mendelian Errors had errors at just 0.84% of SNPs. Further, 60% of SNPs used to construct the PGI had R^2^ INFO scores above 0.98, while 96% of SNPs had R^2^ INFO scores above 0.9.

### Measures

#### Parental body mass index.

Parental height and weight were obtained via self-report at 9 months, 3y, 5y, and 7y sweeps. Weight was asked of fathers and non-pregnant mothers only, and mothers were additionally asked at 9 months for their weight pre-pregnancy. To avoid retrospective measurement error and to maximise sample size, we defined BMI (kg/m^2^) for each parent using their first available, contemporary (i.e., post-pregnancy) report. Correlations between sweeps were ~ 0.85 or greater. Most observations (91%) were drawn from the 9 months sweep.

#### Offspring adiposity.

Child’s height and weight were obtained at ages 3y, 5y, 7y, 11y, 14y, and 17y via direct measurement by interviewers. We converted these to BMI (kg/m^2^), which we used as our primary measure. Nevertheless, as BMI is an imperfect measure of adiposity, particularly among children [39], we supplemented it with several other adiposity-related measures. First, as BMI changes rapidly with age during childhood and adolescence, we converted raw BMI to age and sex-adjusted z-scores using growth reference charts (the 1990 UK reference panel) [40] with the *childsds* package in R [41]. This procedure projects observed BMI onto the distribution of BMI in the reference sample, which has been transformed to have a standard normal (mean = 0; SD = 1) distribution. Second, we used height and weight (z-scores) separately to examine whether associations were related to weight, in particular; BMI is not independent of height in children and adolescents [39]. Third, we examined if results were similar when using direct measures of fat mass collected at ages 7y, 11y, 14y, and 17y [42]: body fat percentage, ratio of fat mass to fat free mass, fat mass index, and fat-free mass index. Fourth, we used waist-to-height ratio measured at 5y and 7y. Further details on these variables are provided in the S1 Text.

Finally, to test for intrauterine effects specifically, we used birth weight (grams), collected at 9 months or 3y from parents who consulted their child’s *red book* (a portable health record), where available.

#### Offspring diet.

Child dietary data were collected at each sweep of the MCS via reports from parents or cohort members. At ages 3y, 5y, 7y and 11y, the parent with the main responsibility for caring for the child (typically the child’s mother) was asked for the frequency or amount of consumption of various foodstuffs and drinks, with the specific questions asked changing between sweeps. At ages 14y and 17y, dietary questions were asked to cohort members instead.

From the dietary questions, we created separate variables for consumption of fruit (5y, 7y, 11y, 14y and 17y), vegetables (14y and 17y), fruit or vegetables (3y), fast food (14y and 17y), sugary drinks (11y, 14y, and 17y), and artificially sweetened drinks (11y, 14y, and 17y). Except for fruit or vegetable consumption at 3y, which is a binary variable, each of these was an ordered categorical variable with 3 or more response categories.

As self-report dietary data is captured with measurement error [43], we used Multiple Correspondence Analysis (MCA) to extract a latent factor for each sweep capturing common variation among the individual dietary items. As only one diet question was asked at 3y, 5y and 7y, we could only perform MCA from 11y onwards. We standardized the diet factor variables (mean = 0, SD = 1) and coded all diet variables such that higher values indicated healthier diet (e.g., greater consumption of fruit and veg, lower consumption of sugary drinks and fast food, etc.). Full details on the dietary measures, including factor loadings, are provided in the S1 Text.

#### Polygenic indices for body mass index.

We created PGIs for mothers, fathers and children based on summary statistics from one of the largest GWAS of adulthood BMI currently available (n =~ 700,000; sex adjusted results from 18). We constructed the PGIs using PRSice-2 [44], disregarding ambiguous alleles and assuming additive genetic effects. We used clumped, genome-wide significant hits (p < 5e-8, R^2^ < 0.01, 1,000 kb window). The final PGI scores were based on 1,075 SNPs. For interpretability, we scaled the PGIs to have mean = 0 and standard deviation = 1 in the complete trio sample.

#### Covariates.

We included variables for the child’s sex and age at BMI assessment (modelled with two natural splines) [45], maternal age at birth, maternal years of education, family socioeconomic class (five category National Statistics Socio-economic Classification; NS-SEC), and (child’s) 10 genetic principal components (PCs). Genetic PCs were used to account for population stratification. Mother’s education and family socioeconomic class were included as potential confounders that may explain differences in child BMI and diet not directly related to parental BMI. Both were measured at age 9 months, or, if missing, at the next sweep at which data were available. Family socioeconomic class was measured as the higher of the resident caregivers’ occupational class.

### Statistical analysis

The primary aims of the analysis were to (a) examine the association between parental BMI and offspring adiposity and diet accounting for direct genetic transmission and (b) to compare this with associations obtained with the standard multivariable regression approach that does not account for genetic inheritance and relies upon phenotypic data only.

To address (a), we ran a series of multivariable Mendelian Randomization (MR) models using instrumental variables two stage least squares regression (IV-2SLS). Mother’s and father’s BMI were instrumented with parental PGIs also conditioning upon offspring PGI and the covariates listed above. Conditioning upon offspring PGI allowed us to isolate indirect from direct genetic effects (a DAG representing the analysis is shown in Fig 1b). The MR models were repeated for each outcome variable and age of assessment. We analysed each sweep separately as, based on previous results [22,25], we anticipated effect sizes to differ by age, but had no *a priori* expectation as to the form this variation would take (e.g., linear change). For individual diet variables, we first dichotomized these and used linear probability models for the second stage regressions (see S1 Text for categories used for dichotomization).

BMI is a complex trait, determined by several other traits under genetic influence and likely picked up by the PGI – PGIs based on the Yengo et al. [18] GWAS used here, or other GWAS of BMI [e.g., 46] are related to several phenotypes upstream of adiposity, such as physical activity [47], (disordered) eating behaviour [48–50], and food parenting practices [51] (though parenting practices may reflect responses to offspring genotype [52]). The MR analysis aimed to assess whether direct genetic transmission could explain correlations between parent and offspring BMI: residual associations of the parental PGIs and offspring BMI, after adjustment for offspring PGI, may be due to pleiotropic effects that are not mediated via parental BMI.

To address (b), we regressed offspring adiposity and diet variables upon parental phenotypic BMI, again repeating models for each outcome variable and age of assessment – parental phenotypic BMI was not instrumented and instead entered into models directly. We again adjusted for the covariates listed above, but did not control for offspring PGI following standard practice. To compare estimates from MR and phenotypic regression models, we calculated the difference in coefficients between models, obtaining confidence intervals using bootstrapping accounting for complex survey design (Rao and Wu method, 500 bootstrap samples) [53,54].

As a further analysis, we estimated multivariable regression models using parental PGIs (instead of parental phenotypic BMI) directly as including mother’s, father’s and child’s PGIs in models simultaneously allowed us to assess the relative size of direct (child’s PGI) and indirect (mother’s and father’s PGIs) genetic effects upon offspring adiposity and diet.

To ensure results were not driven by outliers, we deleted values which were three or more standard deviations away from the (sweep-specific) sample mean. To maximise power, we used regression-specific complete case data. Sample sizes ranged 1,653-2,609. Sample sizes differed across analyses due to missing data for outcome variables or covariates, loss to follow-up, death, or emigration. As the level of selection into the genotyped sample was high (trio data available for < 20% of the eligible sample), we constructed non-response weights for use in our analyses; further detail on the construction of these weights is provided in the S1 Text. Given the MCS uses a complex stratified survey design, we combined these weights with sample recruitment weights to attempt to make the sample representative of the population from which MCS was sampled.

In sensitivity analyses, we used multiply imputed data instead of complete cases. We imputed to two samples (again using genetic non-response weights): the set of families with genotyped trios and the set of families with genotyped duos (mother-offspring or father offspring pairs; n = 5,911; 38.2% of total European ancestry sample). We also repeated models dropping father’s PGI and using observations from genotyped mother-offspring pairs (n = 5,357; 34.7% of the total European ancestry sample) as this allowed us to double the sample size compared to the genetic trios and thus obtain more precise estimates. We additionally descriptively examined whether missingness and attrition were related to outcome variables and participant characteristics to assess the likely degree and direction of bias. As using a weighted PGI may inflate Type I error rates [55], as a final sensitivity analysis, we repeated main analyses using an unweighted PGI instead [18].

Data cleaning and analyses were performed using R version 4.3.1 [56], with the *survey* [57] and *srvyr* [58] packages used to account for MCS’s complex survey design. The code used to run the analysis is available at https://osf.io/5vfnq/.

### Role of the funding source

The funders had no role in study design, data collection and analysis, decision to publish, or preparation of the manuscript. All researchers listed as authors are independent from the funders and all final decisions about the research were taken by the investigators and were unrestricted.

## Results

### Descriptive statistics

**Fig 2:**
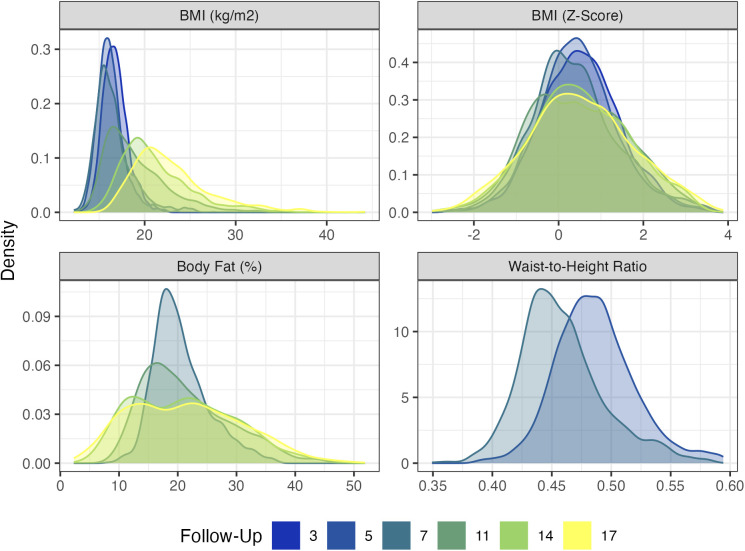
Distribution of four adiposity measures by survey sweep. Weighted with recruitment weights.

The mean, variation, and skewness of BMI increased as the cohort aged (Fig 2). The diet MCA factor variable was highly variable across sweeps and only weakly correlated with contemporary child BMI z-scores (-0.05 < ρ < 0.02; Fig A in S1 Text). Descriptive statistics for the individual diet variables are shown in Fig B in S1 Text.

The PGIs were correlated with several measured confounders (Table 1). Higher levels of each PGI were associated with less advantaged family social class, fewer years of maternal and paternal education, and earlier maternal or paternal age at birth. However, conditioning upon parental PGIs, child’s PGI was only associated with paternal and maternal age at birth (p < 0.05). Maternal and paternal BMI were weakly correlated (ρ = .21) and the correlation between mother’s and father’s PGIs was ρ = -.02. Each parent’s PGI was also unrelated to the other parent’s BMI (i.e., father’s PGI was not related to mother’s BMI). Parental PGIs were strong instruments for parental BMI (F-statistics > 35 in each case).

**Table 1:** Descriptive Statistics. Association between PGI and covariates by person (child, mother or father). Genetic trio sample. For continuous covariates, figure shown is correlation between PGI and covariates. For categorical covariates, figures shown are mean (SD) of PGI in given category. Wald tests were performed to examine whether covariates were related to PGI. * p < 0.05; ** p < 0.01; *** p < 0.001.

Variable		PGI		
		Child	Mother	Father
	Birthweight (g)	0.02	0.05	-0.02
Sex	Male	0.02 (0.98)	-0.01 (1.06)	0.02 (1.01)
	Female	0 (0.98)	-0.06 (0.95)	0.02 (1.01)
	Gestation Age (Months)	-0.03	-0.02	-0.04
	Maternal Age at Birth	-0.11***	-0.09***	-0.03
	Paternal Age at Birth	-0.1***	-0.07*	-0.05
	Mother’s BMI	0.13***	0.23***	0.02
	Father’s BMI	0.1***	0.01	0.23***
Family social class (NS-SEC)	Managerial and Professional	-0.05 (0.98)**	-0.11 (1) **	-0.01 (1.01)*
	Intermediate	-0.07 (0.98)	-0.12 (1.09)	-0.06 (0.99)
	Small Employer and Self-Employed	-0.04 (1.02)	-0.07 (0.95)	-0.12 (1.04)
	Lower Supervisory & Technical	0.19 (1)	0.2 (1.03)	0.15 (0.97)
	Semi-Routine and Routine	0.2 (1)	0.08 (0.94)	0.16 (1.05)
	Not Working	0.07 (0.92)	0.07 (0.99)	0.07 (0.96)
	Mother’s Years of Education	-0.08**	-0.09***	-0.05*
	Father’s Years of Education	-0.08***	-0.07**	-0.08***
Country of Birth	England	-0.03	-0.05	0.01
	Wales	0.02	0.05	-0.01
	Scotland	0.02 (0.99)	-0.03 (1.01)	0.03 (1.01)
	Northern Ireland	0.05 (0.98)	-0.01 (1.05)	0.11 (0.99)

### Parental BMI and offspring adiposity

**Fig 3:**
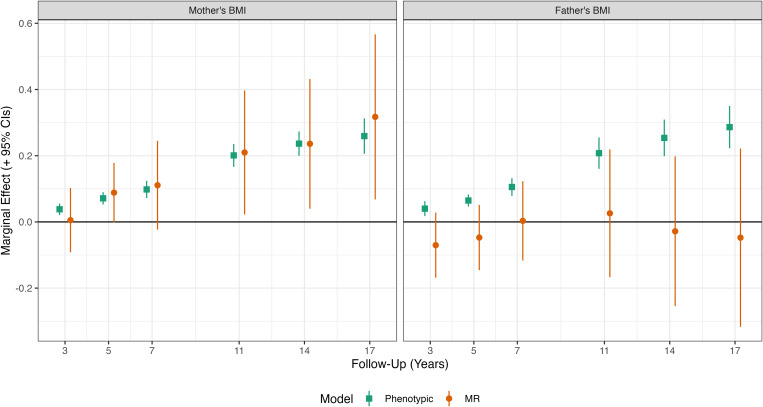
Relationship between mother’s and father’s BMI and offspring BMI by survey sweep estimated by phenotypic linear regression and Mendelian randomization. Results show the kg/m^2^ difference in offspring BMI due to +1 kg/m^2^ increase in the relevant parent’s BMI. Derived from Mendelian Randomization (MR; IV 2SLS) and (phenotypic) multivariable regression of BMI on mother’s and father’s BMI, with adjustment for sex, age at follow-up (two natural splines), maternal age at birth, family social class, mother’s education years, and 10 genetic principal components. In MR analysis, parental BMI instrumented with mother’s and father’s PGI and additionally adjusted for offspring PGI. Regressions were weighted with non-response weights to account for selection into the genotyped study sample.

Mother’s and father’s BMI were consistently related to higher child BMI in standard (phenotypic) multivariable regression models (Fig 3; see Table A in S1 Text for full regression results). Associations were stronger at older ages and similar in size – on the absolute scale – for mother’s and father’s BMI. A 1 kg/m^2^ increase in mother’s BMI was associated with a 0.26 kg/m^2^ (95% CI = 0.21, 0.31) increase in child’s BMI at age 17y. The corresponding figure for father’s BMI was 0.29 kg/m^2^ (95% CI = 0.22, 0.35). However, mother’s BMI showed greater sample variability than father’s BMI (4.7 SD vs 3.9 SD, respectively). A 1 SD unit increase in mother’s BMI was therefore related a larger increase in offspring BMI than a 1 SD unit increase in father’s BMI was, though confidence intervals overlapped. (Bivariate Pearson’s correlations between parental BMI and offspring BMI are shown in Table 2).

**Table 2:** Pearson’s Correlation Between parental BMI and offspring BMI, by offspring age and parent. Genetic trio sample weighted for non-response.

Follow-Up	Mother x Offspring	Father x Offspring	Difference
3	0.15 (0.1, 0.21)	0.14 (0.08, 0.2)	-0.01 (-0.08, 0.06)
5	0.27 (0.22, 0.32)	0.22 (0.17, 0.26)	-0.05 (-0.12, 0.01)
7	0.27 (0.22, 0.32)	0.24 (0.18, 0.29)	-0.04 (-0.1, 0.02)
11	0.34 (0.3, 0.39)	0.29 (0.24, 0.34)	-0.05 (-0.12, 0.01)
14	0.35 (0.3, 0.4)	0.30 (0.23, 0.36)	-0.06 (-0.12, 0.01)
17	0.33 (0.28, 0.39)	0.29 (0.23, 0.36)	-0.04 (-0.11, 0.03)

Mother’s BMI remained consistently related to higher child BMI in MR analysis (left plot, Fig 3). Effect sizes were similar to those obtained using phenotypic multivariable regression; tests comparing coefficients across phenotypic and MR models showed no clear evidence of differences at any age. However, estimates from MR models were less precise than those from phenotypic models. In the MR model, a 1 kg/m^2^ increase in mother’s BMI was associated with a 0.32 kg/m^2^ (95% CI = 0.07, 0.57) increase in child’s BMI at age 17y.

Point estimates for the association between father’s BMI and child’s BMI in MR models were smaller in size and typically close to the null. For instance, at age 14y, a 1 kg/m^2^ increase in father’s BMI was associated with a -0.03 kg/m^2^ (95% CI = -0.25, 0.20) increase in child’s BMI. Z-tests showed clear evidence that associations obtained for father’s BMI in MR models were smaller than those using phenotypic multivariable regression (p < 0.05 at ages 3y, 14y and 17y).

Regarding alternate measures of adiposity, mother’s and father’s BMI were consistently related to child’s BMI z-scores and weight z-scores but (for mother’s BMI) not height z-scores in multivariable regression models (Fig C in S1 Text). Again, associations for mother’s BMI from MR models were broadly consistent with those from multivariable regression models, while associations for father’s BMI were closer to the null. Mother’s and father’s BMI were consistently related to waist-to-height ratio, fat mass, and fat-to-fat-free ratio in phenotypic multivariable regression models. There was little strong evidence of association in MR models, though point estimates for mother’s BMI were consistent with effects on increasing adiposity (Fig D in S1 Text).

Mother’s BMI was positively related to child’s birthweight in both (phenotypic) multivariable and MR models (Table A in S1 Text). The phenotypic association was 15.18 grams (95% CI = 9.61, 20.75) per 1 kg/m^2^ increase in mother’s BMI, while the association observed in MR models was 24.02 grams (95% CI = -5.48, 53.52) per 1 kg/m^2^. There was little evidence that father’s BMI was associated with child’s birthweight in either phenotypic or MR regression models. Corresponding figures were 2.38 grams (95% CI = -4.34, 9.11) and -16.18 grams (95% CI = -44.81, 12.46), respectively.

### The association between parental BMI and child diet

**Fig 4:**
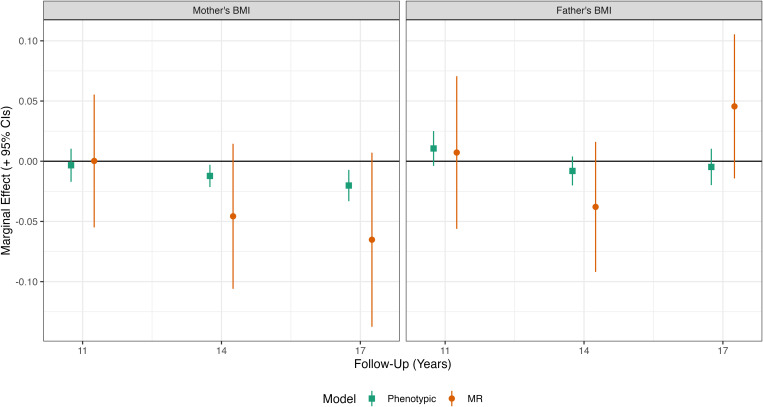
Mother’s and father’s BMI and offspring diet by survey sweep. Results show the SD difference in offspring diet factor score due to +1 kg/m^2^ increase in the relevant parent’s BMI. Derived from Mendelian Randomization (MR; IV 2SLS) and ‘phenotypic’ multivariable regressions of offpsring on mother’s and father’s BMI, with adjustment for sex, age at follow-up (two natural splines), maternal age at birth, family social class, mother’s education years, and 10 genetic principal components. In MR analysis, parental BMI instrumented with mother’s and father’s PGI with additional adjustment for child’s PGI. Offpsring diet captured extracting a factor from multiple diet items using MCA. Regressions were weighted with non-response weights to account for selection into the genotyped study sample.

Mother’s BMI was inversely related to the child diet MCA factor in (phenotypic) multivariable regression models at ages 14y and 17y, indicating a less healthy diet for children whose mothers had higher BMI (Fig 4, left panel; see Table A in S1 Text for full regression results). Effect sizes were small, however: a 1 kg/m^2^ increase in mother’s BMI was related to a -0.02 SD (95% CI = -0.03, -0.01) lower child diet factor score at age 17y. Point estimates were larger (in absolute terms) at 14y and 17y in MR than phenotypic regression models, though effects sizes were again small and confidence intervals overlapped the null. The MR model predicted a 1 kg/m^2^ increase in mother’s BMI was related to a -0.07 SD (95% CI = -0.14, 0.01) lower child diet factor score at age 17y.

There was little evidence of an association between mother’s BMI and child diet factor score at age 11y in either multivariable or MR models. Father’s BMI was not consistently related to child’s diet factor scores, though there was evidence of association at age 14y in both multivariable and (less precisely estimated) MR models: MR results suggested a 1 kg/m^2^ increase in father’s BMI was related to -0.04 SD (95% CI = -0.09, 0.02) lower diet factor score at age 14y.

Looking at individual diet items, there was evidence from MR models that mother’s BMI was related to greater frequency of fast food consumption, greater consumption of sweetened drinks (ages 11y and 14y), and lower consumption of fruit (left column, Fig E in S1 Text). There was little clear association between father’s BMI and any individual child diet items in MR models (right column, Fig E in S1 Text).

### Comparison of direct and indirect genetic effects

**Fig 5:**
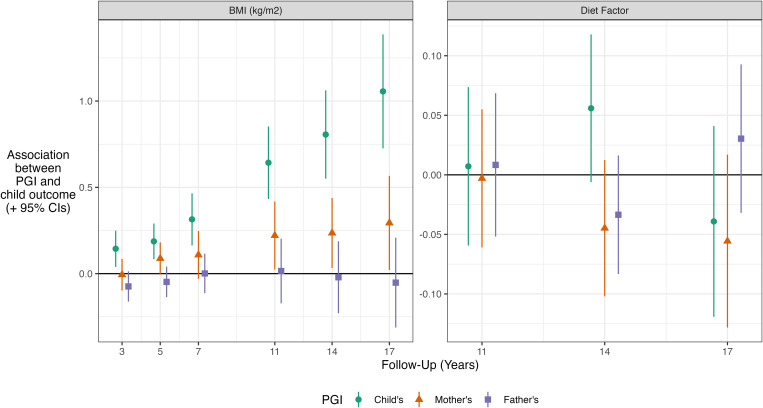
Association between parental and offspring PGI and offspring BMI z-score (left panel) and diet (right panel) by survey sweep. Results show the kg/m^2^ difference in offspring BMI due to 1 SD increase in the relevant PGI. Derived from OLS regressions of BMI z-scores (by sweep) on mother’s, father’s and child’s adulthood PGI, with adjustment also for sex, age (two natural splines), maternal age at birth, family socioeconomic class, mother’s education year, and 10 genetic principal components. Regressions were weighted with non-response weights to account for selection into the genotyped study sample.

In models examining associations between BMI and child’s and parent’s PGI, from age 5y onwards, the (conditional) association between mother’s PGI and offspring BMI was approximately one-quarter to one-half the size of the association found for offspring’s PGI (left panel, Fig 5; see Table B in S1 Text for full regression results). This indicated that maternal indirect genetic effects comprise a substantial component of total genetic effects (indexed by the PGI) for this phenotype. Conditional associations between the father’s PGI and child BMI were small at each follow-up and confidence intervals overlapped the null.

The association between the mother’s PGI and birthweight (20.11 g, 95% CI = -8.73, 48.95, per 1 SD increase in the mother’s PGI) was larger than the corresponding direct genetic effect (14.71 g, 95% CI = -17.86, 47.28), though confidence intervals overlapped. Further, there was little evidence of an association between father’s PGI and birthweight (-13.27 g, 95% CI = -39.42, 12.87, per 1 SD increase in father’s PGI)

For child’s diet, mother’s PGI was associated more strongly with less healthy offspring diet than the child’s PGI was (right panel, Fig 5), though confidence intervals overlapped. There was no clear or consistent association between child’s PGI and diet. In fact, at age 14y, offspring’s PGI was related to a *more healthy* diet, though confidence intervals overlapped the null – a 1 SD in offspring’s PGI was associated with 0.06 SD (95% CI = -0.01, 0.12) increase in the diet factor variable.

### Sensitivity analyses

MR estimates were qualitatively similar when not including father’s PGI and BMI in models, both within the genetic-trio sample (middle vertical panel, Fig F in S1 Text) and in the larger sample of genotyped mother-offspring pairs (right panel, Fig F in S1 Text). MR estimates in the mother-offspring pairs were typically smaller in size, however (albeit to a limited extent). Further, in the mother-offspring pair sample, confidence intervals for the association between maternal BMI and offspring diet overlapped the null (bottom right hand panel, Fig F in S1 Text).

MR estimates were qualitatively similar for both maternal and paternal BMI when using multiple imputation (Figs G and H in S1 Text). Point estimates for the effect of maternal BMI upon offspring BMI, diet and birthweight were smaller in the sample with genotyped mother-offspring or father-offspring pairs than in the genetic trio sample, though confidence intervals overlapped.

Results were consistent when using an unweighted PGI instead of a weighted PGI (Fig I in S1 Text): mother’s BMI, but not father’s BMI, was related to offspring BMI in MR models, with the association increasing into adolescence. However, associations with the unweighted PGI were less precisely estimated, and confidence intervals overlapped the null.

### Assessment of sample bias

Compared to eligible participants without genetic trio data, those with genetic trio data were more likely to have healthy diets, higher birthweight, an older mother, parents with greater BMI, and a more advantaged socioeconomic background (Table C in S1 Text, Column 2). There was less bias according to these factors among the sample of genotyped mother-offspring pairs, though differences remained (Table C in S1 Text, Column 3). Higher BMI z-scores were related to a lower chance of participation at subsequent sweeps (Fig J in S1 Text). For instance, a 1 SD higher BMI z-score at age 11y was related to ~ 3.4% points lower probability of participating at 17y.

## Discussion

Using lifecourse data from over 2,600 genetic trios in the Millennium Cohort Study, we found evidence that mother’s and father’s BMI were associated longitudinally with offspring adiposity between ages 3y to 17y. Associations increased in size as the children aged. However, our results suggested that the association between father’s and child’s BMI was explained by direct genetic inheritance: there was little evidence of an association in MR models that accounted for transmission of adiposity related-genetic variants. Mother’s BMI was associated with offspring BMI in MR models, consistent with the effect of maternal genes operating partly indirectly via maternal traits. Indirect genetic effects were estimated to be between 20–50% of direct genetic effects for offspring BMI. Alternate measures of adiposity, such as fat mass and waist-to-height ratio, showed positive, but imprecise, associations with maternal BMI when accounting for genetic inheritance. There was weak evidence of an association between mother’s BMI and child’s diet, at least at ages 14y and 17y, and again when direct genetic inheritance was accounted for.

Our results are (partly) consistent with analyses of a regional UK birth cohort which find associations between maternal adiposity and offspring BMI in adolescence [22,27]. Our results differ in finding evidence of association in earlier childhood. Further, effect sizes (when converted to SD units) were typically larger (by 50% or more) in the present study. If causal, our results suggest that intervening to reduce maternal BMI while the child is in utero could have modest long-term intergenerational effects upon obesity rates.

To our knowledge, our analysis of father’s BMI using MR is novel in the literature. The finding that father’s BMI is not associated with offspring BMI after accounting for direct genetic inheritance may explain the small effect sizes found by Kong et al. [23] who did not separate maternal and paternal effects. However, their study also differed in measuring offspring BMI in young adulthood – parental effects could be larger in childhood and adolescence when individuals are more likely to live with their parents. Results require replication in an older sample.

We found little evidence of substantial associations between father’s BMI and offspring’s BMI (conditional on genotype). This is intriguing in light of the large number of studies investigating father-child correlations in phenotypic BMI [4,6,59]. Given precision in the present study, replication is required. If the present results are replicated, they suggest phenotypic correlations between father’s BMI and offspring’s BMI could be explained by direct genetic inheritance and genetic confounding. However, it should be noted that the MR analysis estimated a Local Average Treatment Effect (LATE) – in this case, the effect of the variation in (parental) BMI induced by the PGI. This may explain the qualitative difference between results in phenotypic and MR models. Nevertheless, future observational studies that test for environmental causes of the father-child adiposity correlations should consider using genetically informed designs, including designs subject to different assumptions than here, such as adoption studies. It is also possible that paternal indirect genetic effects are stronger in contexts where fathers are more involved in childcare – future studies could examine genetic nurture in countries marked by greater gender equality.

In contrast to fathers, we found little evidence that direct genetic inheritance biased phenotypic estimates of maternal effects on offspring adiposity. Associations were little changed when we included or omitted father’s PGI and parental PGIs displayed little correlation. This is promising given that, for data availability reasons, there are typically many more mother-child pairs than full trios in existing genetic studies (e.g., due to study design, cost constraints, or parental separation).

Our finding of some evidence that mother’s BMI was related to higher offspring birthweight was in line with other MR studies [22,60]. Maternal effects at later ages may operate via prenatal influence. However, in MR models, there was also some (weak) evidence that mother’s BMI was related to the offspring’s diet, at least at some ages, which is also consistent with a postnatal pathway. Nevertheless, dietary data were imprecisely measured – we used few measures at each sweep and these were captured via self-report using high-level response categories. Parental practices may have influenced offspring diet in subtle ways which are challenging to capture in population studies (e.g., relatively small increases in total calories or fat content which cumulatively impact offspring weight gain) [61]. The diet MCA factor variable also had little correlation with observed BMI. Future studies should use more robust measures of diet as well as examine other potential pathways, such as physical activity.

Additional work is also required on the specific parental behaviours and traits which may explain associations. While we were able to account for direct genetic transmission with our study design, the BMI PGI is associated with several other phenotypes that may drive associations via horizontal pleiotropy, rather than reflecting a causal effect of parental BMI, per se (i.e., the MR exclusion restriction assumption is likely violated). Notably, the PGI we used is associated with eating behaviour [48–50], which is upstream of parental BMI; parents may influence offspring adiposity through factors related to this (e.g., availability of unhealthful foodstuffs in the home).

Twin studies suggest that the total effect of the shared environment upon BMI decreases from childhood to adolescence [62]. Our results are consistent with maternal genetic nurture being one component of the shared environment. While our study found that the estimated effect of maternal BMI upon offspring BMI was relatively uniform (on the relative – i.e., SD – scale) across childhood and adolescence, it is possible that other aspects of the shared environment become less important as children age.

The study design used here can also be applied to examine the role of other genetically influenced parental traits. For instance, father’s education or cognitive ability could have greater effects on the food and wider social environments the child is raised within (e.g., access to healthy foods and social norms regarding body shape). Though Kong et al. [23] find no evidence for genetic nurture effects on parental BMI, they do observe genetic nurture effects on offspring BMI when using a PGI for educational attainment, specifically. The role of other traits may be investigated using a similar MR approach to that used here but could also involve GWAS of BMI that separate direct and indirect genetic effects, either through explicit study design (e.g., GWAS of trios) or through summary data methods such as genomicSEM [63].

### Strengths and limitations

This study had a number of strengths. We used a PGI based on data from a large GWAS of adult BMI [18]. Previous null results may be due to less powerful PGIs used [22]. We used data on complete genotyped parent-offspring trio data which allowed us to better account for bias due to assortative mating and to compare maternal and paternal indirect genetic effects. Further, our data were drawn from a nationally representative cohort with multiple measurements of offspring diet and other adiposity-related traits. Data collection was also relatively recent and, as such, particularly relevant for the current obesity epidemic.

However, our sample was selected, with genetic trio data only available for 45% of those observed with resident parents at age 14y, reflecting the practical challenges of obtaining genetic data in nationally representative samples of families. Biological parental figures not present in the household were also not included. However, the target population for this analysis is arguably those parental figures who are closely involved in offspring development and growth. It should also be noted that the sample is likely more representative than several other datasets used widely in the genetics literature (e.g., UK Biobank).

Though we constructed and used non-response weights, there was still evidence of attrition bias, with high levels of BMI related to a greater likelihood of dropping out of the study. This may have attenuated associations towards the null. While our analytical design allowed us to account for genetic inheritance, the MR approach estimates a LATE and, further, estimates can still be biased by other factors, such as horizontal pleiotropy and confounding by social or familial factors. Notably, parental BMI PGIs were related to family socioeconomic position. Nevertheless, confounding factors are likely to have biased both maternal and paternal indirect genetic effect estimates and are thus unlikely to explain the differences in effect estimates between parents. This logic also applies to bias arising from a selected sample.

While offspring BMI and adiposity were measured objectively, diet was measured with only a few high-level, self-report survey items. These did not appear to have optimal psychometric properties: diet was very weakly related to BMI and the correlation between diet variables across sweeps was low. A further limitation was the measurement of parental adiposity, which was based on self-reported BMI and was captured postnatally. Self-reported BMI is typically underestimated [64] and BMI does not distinguish fat and lean mass. This may have biased results for fathers, in particular, as BMI is less correlated with fat mass among males [65,66]. Future work should repeat the analysis using direct measures of parental adiposity. A final limitation was that we included the results of many regression models. Some results were inconsistent across ages and may be explained by sample variation.

## Conclusions

Results suggest that maternal BMI may be particularly important for offspring BMI: associations may arise due to both direct transmission of genetic effects and indirect (genetic nurture) effects. Assocation between father’s BMI and offspring BMI were close to the null when accounting for direct genetic transmission. The well replicated (phenotypic) correlation between father’s and child’s BMI may reflect strong genetic confounding. Larger studies are required to confirm these findings.

## Supporting information

S1 Text Including: Fig A: Correlation between diet MCA factor and (a) BMI z-scores and (b) the diet MCA factor itself, by age at follow-up. Fig B: Distribution of individual diet items. Fig C: Association between mother’s and father’s BMI and offspring BMI, weight, and height (z-scores) by survey sweep. Fig C: Association between mother’s and father’s BMI and offspring adiposity by survey sweep. Fig D: Association between mother’s and father’s BMI and offspring diet by survey sweep. Fig E: Association between mother’s BMI and offspring BMI and diet by sample, PGIs used, and survey sweep. Fig F: Association between mother’s and father’s BMI and offspring BMI (z-scores) by sample, PGIs used, and survey sweep. Fig E: Association between mother’s and father’s BMI and offspring diet by sample, PGIs used, and survey sweep. Fig F: Association between mother’s and father’s BMI and offspring BMI by survey sweep and PGI used (weighted or unweighted). Fig G: Difference in probability of participation in given survey sweep according to BMI (z-score) at prior sweep. Table A: Regression Results, Phenotypic and Mendelian Randomization Models. Table B: Regression Results, Association Between PGI and Offspring Phenotypes. Table C: Descriptive statistics by sample. Table D: Description of individual diet variables.
(DOCX)
