# Supplementary material for: The association between parental BMI and offspring adiposity: A genetically informed analysis of trios
Source: PLoS Genet. 2025 Aug 5;21(8):e1011775. doi: 10.1371/journal.pgen.1011775 (PMC12324121; doi:10.1371/journal.pgen.1011775)
Supplement: S1 Text — Including: Fig A: Correlation between diet MCA factor and (a) BMI z-scores and (b) the diet MCA factor itself, by age at follow-up. Fig B: Distribution of individual diet items. Fig C: Association between mother’s and father’s BMI and offspring BMI, weight, and height (z-scores) by survey sweep. Fig C: Association between mother’s and father’s BMI and offspring adiposity by survey sweep. Fig D: Association between mother’s and father’s BMI and offspring diet by survey sweep. Fig E: Association between mother’s BMI and offspring BMI and diet by sample, PGIs used, and survey sweep. Fig F: Association between mother’s and father’s BMI and offspring BMI (z-scores) by sample, PGIs used, and survey sweep. Fig E: Association between mother’s and father’s BMI and offspring diet by sample, PGIs used, and survey sweep. Fig F: Association between mother’s and father’s BMI and offspring BMI by survey sweep and PGI used (weighted or unweighted). Fig G: Difference in probability of participation in given survey sweep according to BMI (z-score) at prior sweep. Table A: Regression Results, Phenotypic and Mendelian Randomization Models. Table B: Regression Results, Association Between PGI and Offspring Phenotypes. Table C: Descriptive statistics by sample. Table D: Description of individual diet variables. (DOCX) [file pgen.1011775.s001.docx]

Supplementary Information

# Measures

## Adiposity

BMI is an imperfect measure of adiposity, particularly among children (Wang et al., 2006). It is intended to be a measure of weight that is independent of height, but this is not case when using the typical formula for BMI (kg/m^2^) in childhood. Thus, we supplemented our analysis with four measures of adiposity collected at ages 7y, 11y, 14y, and 17y following Staatz et al. (2021): body fat percentage, ratio of fat mass to fat free mass, fat mass index, and fat free mass index. Body fat percentage was measured through foot-to-foot bio-electrical impedance analysis using Tanita (Bf-522W) scales by interviewers adhering to standardised protocols. Fat mass (FM) was calculated by multiplying body mass percentage by weight (kg), and fat-free mass (FFM) was calculated as the remainder. To create fat mass and fat-free mass indices, we divided each by height (m^B^) raised to a power (B) such that the resulting indices were independent of height. We calculated B by regressing log FM or log FFM on log height and extracting the coefficient, performing these regressions for each sex and sweep separately. These coefficients are shown in the table below.

| Variable | Sex | 7y | 11y | 14y | 17y |
| --- | --- | --- | --- | --- | --- |
| Fat Mass | Male | 3.98 | 4.76 | 3.00 | 1.95 |
|  | Female | 4.32 | 4.57 | 2.91 | 3.34 |
| Lean Mass | Male | 2.43 | 2.63 | 2.59 | 2.07 |
|  | Female | 2.43 | 2.57 | 2.21 | 1.41 |

## Diet

The individual diet questions, including sweeps collected at, response categories, and corresponding variables from the original dataset, are provided in Table D. We recoded items such that higher values indicated a healthier diet. The correlations between the MCA factors variables and the individual diet measures are displayed below.

| Variable | 11y | 14y | 17y |
| --- | --- | --- | --- |
| Portions Fruit per Day | 0.26 |  |  |
| Freq. Sugary Drinks | -0.07 | 0.65 | 0.65 |
| Freq. Sweetened Drinks | 0.20 | 0.35 | 0.44 |
| Freq. Fast Food |  | 0.66 | 0.66 |
| Freq. Fruit |  | 0.65 | 0.67 |
| Freq. Vegetables |  | 0.67 | 0.68 |

Individual diet items were dichotomised in regression models (full categories were used to derive diet MCA factors). The categories used are displayed in Figure B.

# Construction of Genetic Non-Response Weights

Cohort members (and their parents) who provided valid genetic data were a selected subsample of initial MCS participants. Saliva samples, from which DNA was extracted, were taken at Sweep 6 (age 14y) from cohort members and any resident biological parents. Inclusion in the genotyped trio (mother-father-offspring) sample therefore relied upon cohort members remaining in the study until Sweep 6, the family remaining together at that age, and ability and willingness of family members to give a saliva sample. There was attrition from MCS, movement of parents (typically fathers) from cohort members’ households, and refusal to give DNA samples, so approximately 17% of initial MCS (White European, singleton birth) families provided valid genetic data from offspring, mothers and fathers. The number of mother-offspring pairs was approximately double this (~ 38%) as this did not rely on a co-resident and consenting biological father.

Selection into the genotyped (e.g., genetic trio or mother-offspring pair) samples was not random. To account for potential bias that may arise, we constructed non-response weights which we used in all analyses. We produced bespoke weights for different genotyped samples (trios, duos, child only, etc.) given the difference in sample size across these, but the procedure and variables used to create the weights was the same in each case. Specifically:

1. We first identified several variables with low item missingness that we anticipated being predictive of non-response from prior work in MCS and CLS’ other cohort studies (Silverwood et al., 2024) as well as variables of interest for the current study (e.g., birthweight and parental BMI).
   1. These variables were: sex, date of birth, survey design weight, father’s and mother’s age at cohort member’s birth, gestational age, birthweight, school readiness at age 3y, vocabulary at age 5y, cohort member’s BMI at age 7y, father’s and mother’s BMI, father’s and mother’s years of education, family socioeconomic class, cohort member’s country of birth, response to the specific genotyped sample.
2. Next, we handled (item) missingness in these variables using multiple imputation (MI) via chained equations (20 imputed datasets, burn in = 10).
   1. Continuous variables were imputed with predictive mean matching, binary variables with logistic regression, and other categorical variables with multinomial logistic regression.
   2. All included variables were included as predictors in the individual imputation models.
3. In each imputed dataset, we estimated a logistic regression model of response on the variables listed in 1a. Each variable was included as a main effect with no interaction or non-linear terms added. The target population in this model was any eligible cohort family (White European singleton birth)^[[1]](#footnote-1)^.
4. From each model, we calculated a probability of inclusion in the (specific) genotyped sample for each genotyped family. We then averaged this across imputation datasets and took the inverse to get a genetic non-response weight.
5. As the MCS used a stratified sampling design, we combined this weight (by multiplication) with a design weight supplied with the dataset to make the sample representative of the population from which MCS was drawn. As there was an excess of especially large weights following this step, we truncated the weights at 20.
6. Finally, we normalised the weights so the sum equalled the number of families in the genotyped sample (e.g., for genotyped trios this was 2,630).

# Multiple Imputation Procedure

As a sensitivity analysis, we addressed remaining item missingness in the genotyped samples through multiple imputation (MI). The imputations were performed for three genotyped samples separately: trios (mother-father-offspring; N = 2,630), mother-offspring duos (N = 5,357), and mother-offspring or father-offspring duos (N = 5,911). Imputations were performed in wide format. The variables included in imputation models were: sex, birthweight, mother’s and father’s BMI, mother’s years of education, mother’s age at birth, family socioeconomic class, vocabulary at age 5y, child’s, mother’s and father’s polygenic scores, child’s first twenty genetic principal components, child’s age at Sweeps 1-7 (0y-17y), child’s BMI at Sweep 2-7 (3y-17y), and child’s diet factor scores at Sweeps 5-7 (11y-17y). Age at a specific sweep was only used a predictor for child’s BMI and diet factor scores at the same sweep.

Continuous variables were imputed with predictive mean matching, binary variables with logistic regression, and other categorical variables with multinomial logistic regression. 40 imputed datasets were generated (burn-in = 10). Analyses using the imputed data were pooled using Rubin’s (1987) rules.

# Figures


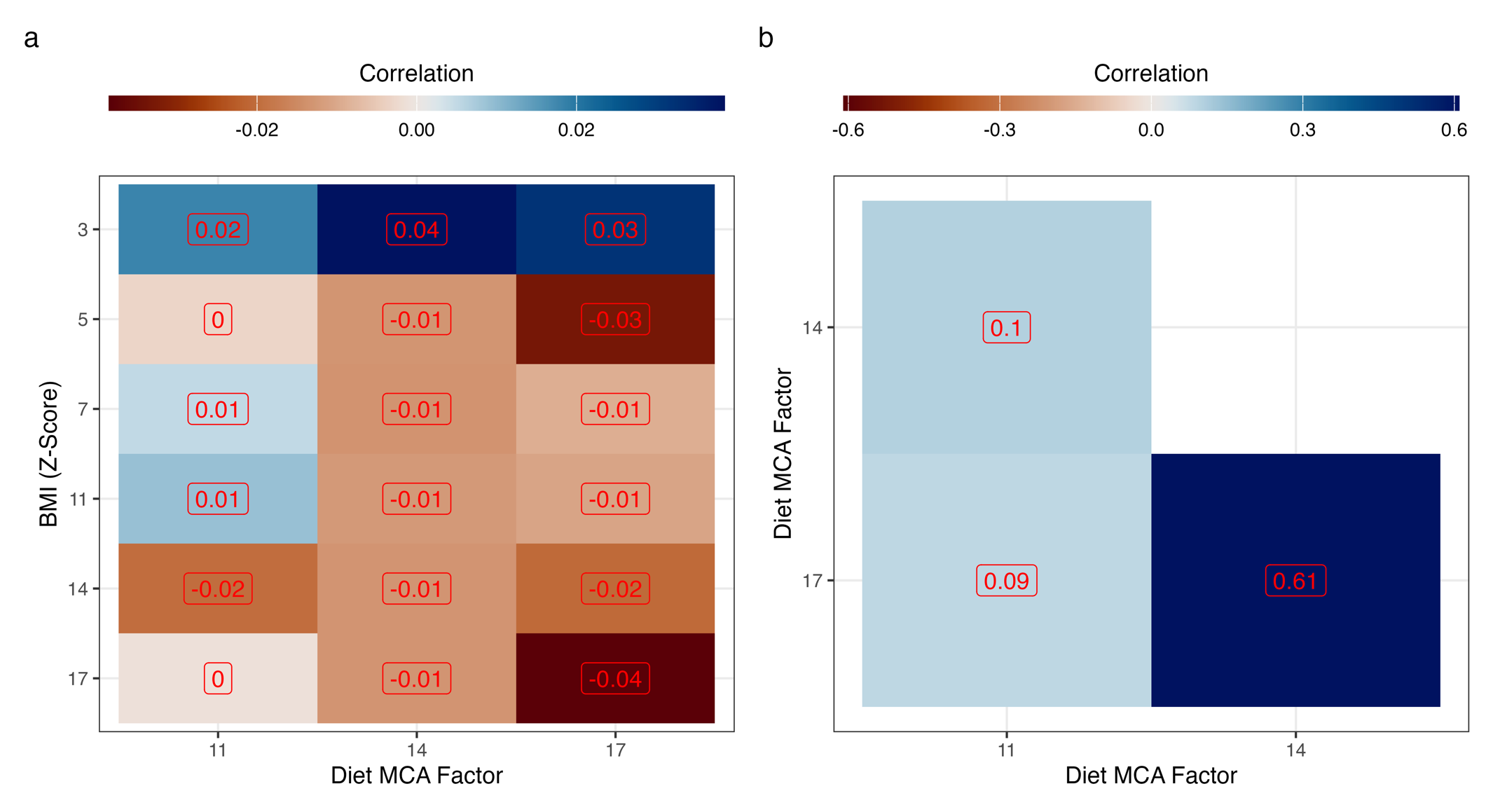


**Figure A: Correlation between diet MCA factor and (a) BMI z-scores and (b) the diet MCA factor itself, by age at follow-up.** Diet MCA factor is coded such that higher values indicate a *healthier* diet. Correlations weighted with recruitment weights and accounting for the cluster stratified sampling design.


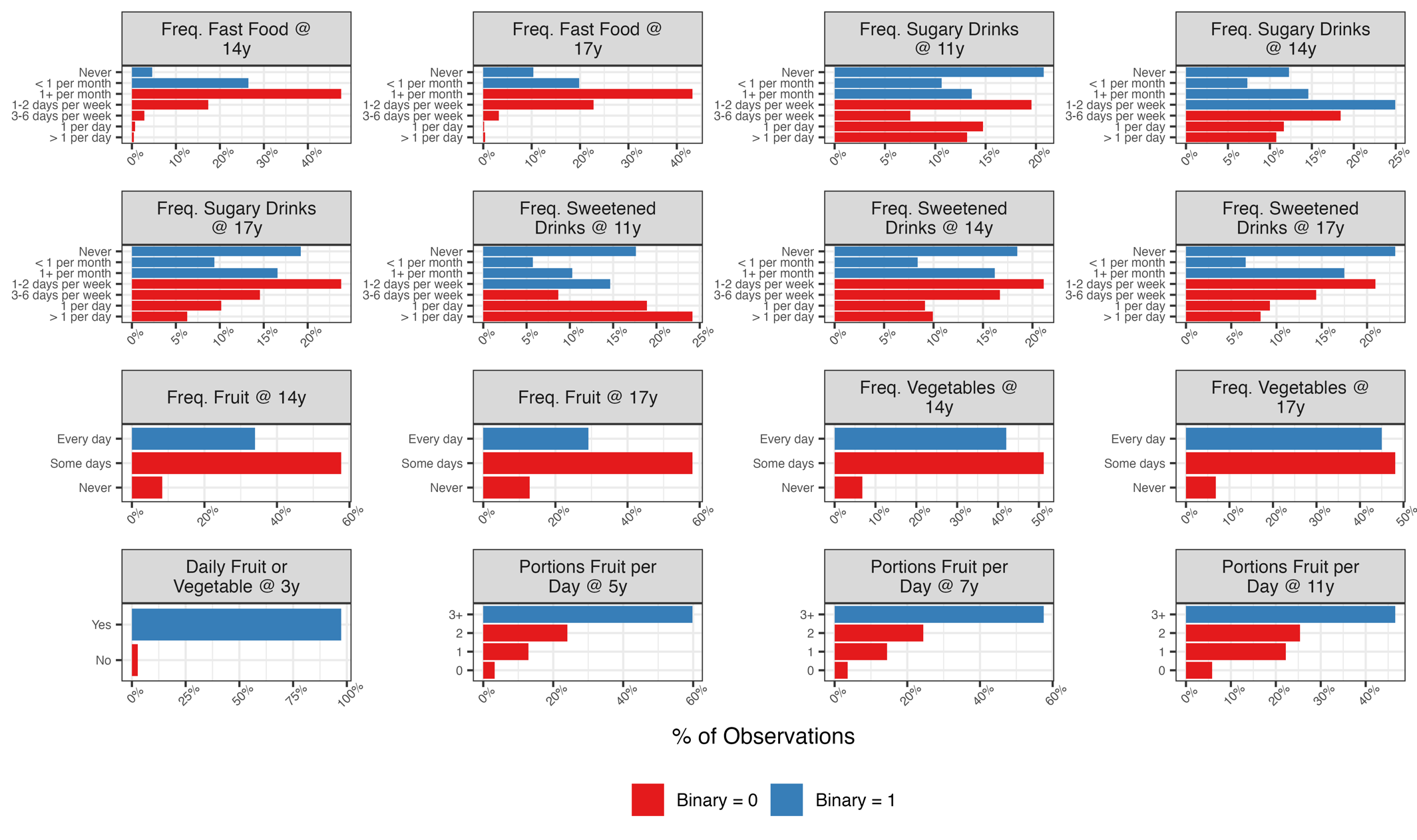


**Figure B: Distribution of individual diet items.** Diet items were dichotomized in regression models to simplify interpretation. The colour of the bar reflects the level the category was dichotomised into (blue = 1, red = 0).


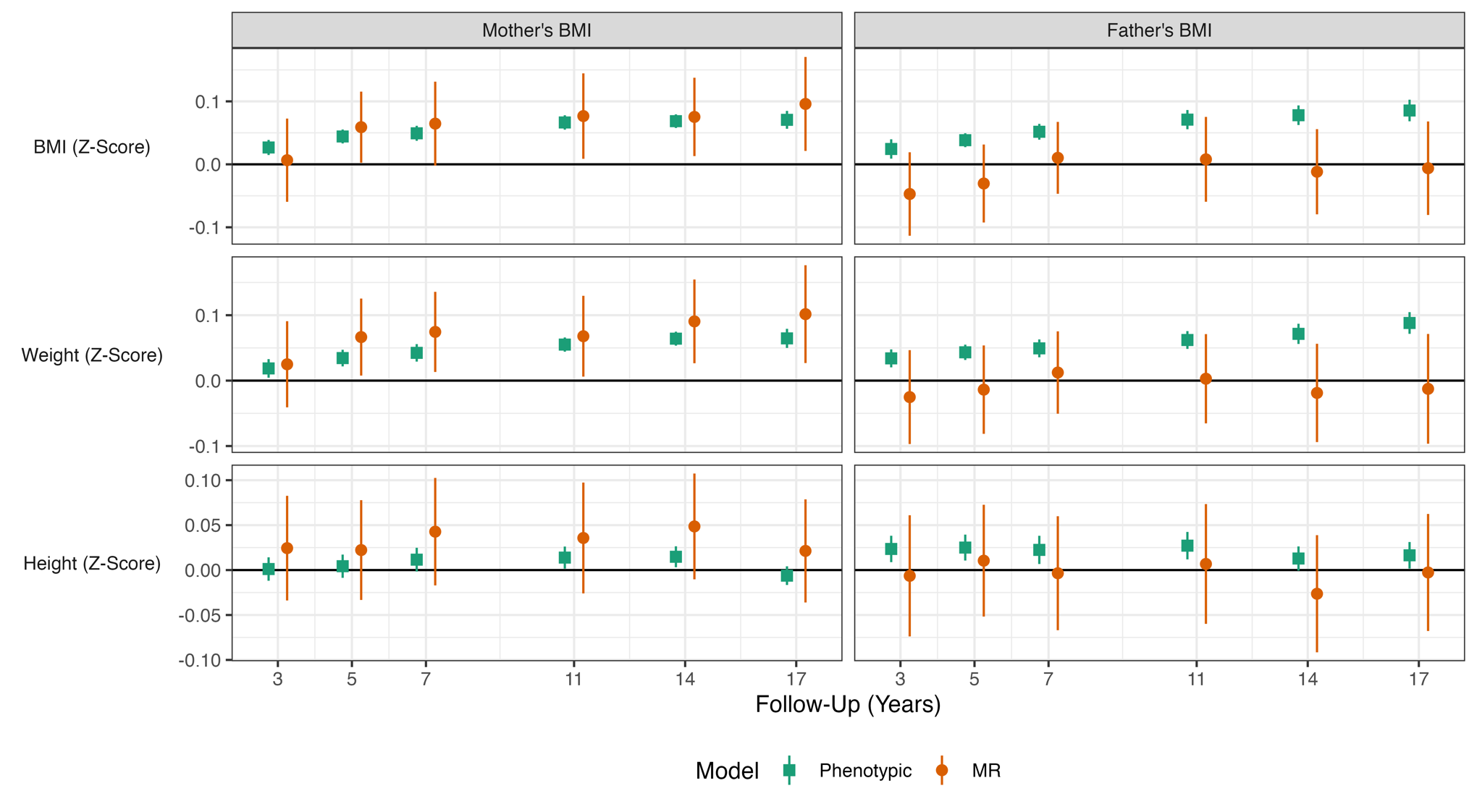


**Figure C: Association between mother’s and father’s BMI and offspring BMI, weight, and height (z-scores) by survey sweep.** Results show the difference in the relevant offspring anthropometric z-score due to +1 kg/m^2^ increase in parental BMI. Derived from Mendelian Randomization (MR; IV 2SLS) and phenotypic multivariable regressions of offspring BMI, weight, and height (z-scores) on mother’s and father’s BMI, with adjustment for sex, age at follow-up (two natural splines), maternal age at birth, family social class, mother’s education years, and 10 genetic principal components. In MR analysis, parental BMI was instrumented with mother’s and father’s PGI and included additional adjustment for offspring PGI. All regressions were weighted with non-response weights to account for selection into the genotyped study sample. Note, BMI z-scores are a non-linear transformation of BMI onto an age-adjusted normal distribution.


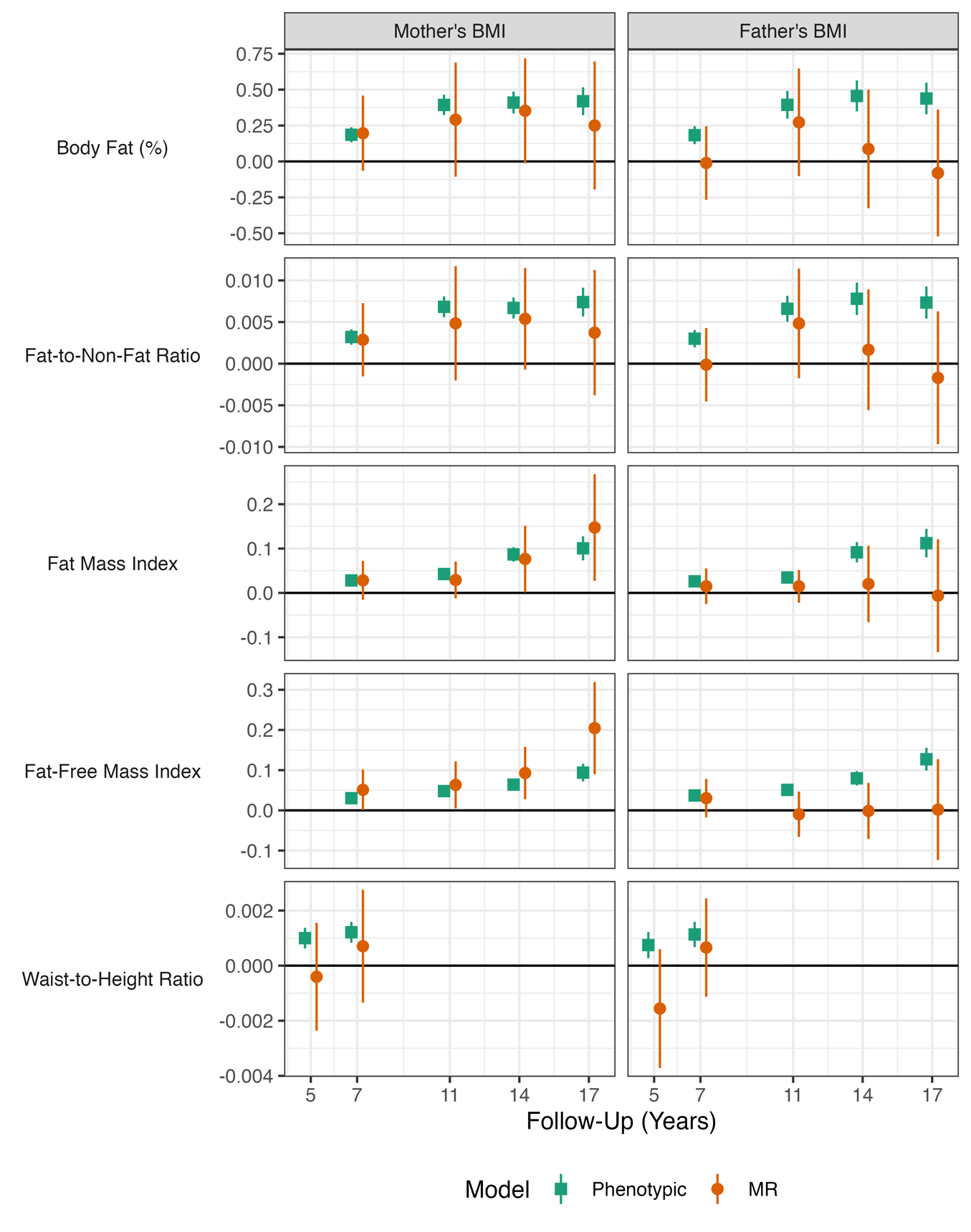


**Figure D: Association between mother’s and father’s BMI and offspring adiposity by survey sweep.** Results show the unit difference in the relevant offspring anthropometric measure due to +1 kg/m^2^ increase in parental BMI. Derived from Mendelian Randomization (MR; IV 2SLS) and phenotypic multivariable regressions of offspring adiposity on mother’s and father’s BMI, with adjustment for sex, age at follow-up (two natural splines), maternal age at birth, family social class, mother’s education years, and 10 genetic principal components. In MR analysis, parental BMI was instrumented with mother’s and father’s PGI and included additional adjustment for offspring PGI. All regressions were weighted with non-response weights to account for selection into the genotyped study sample.


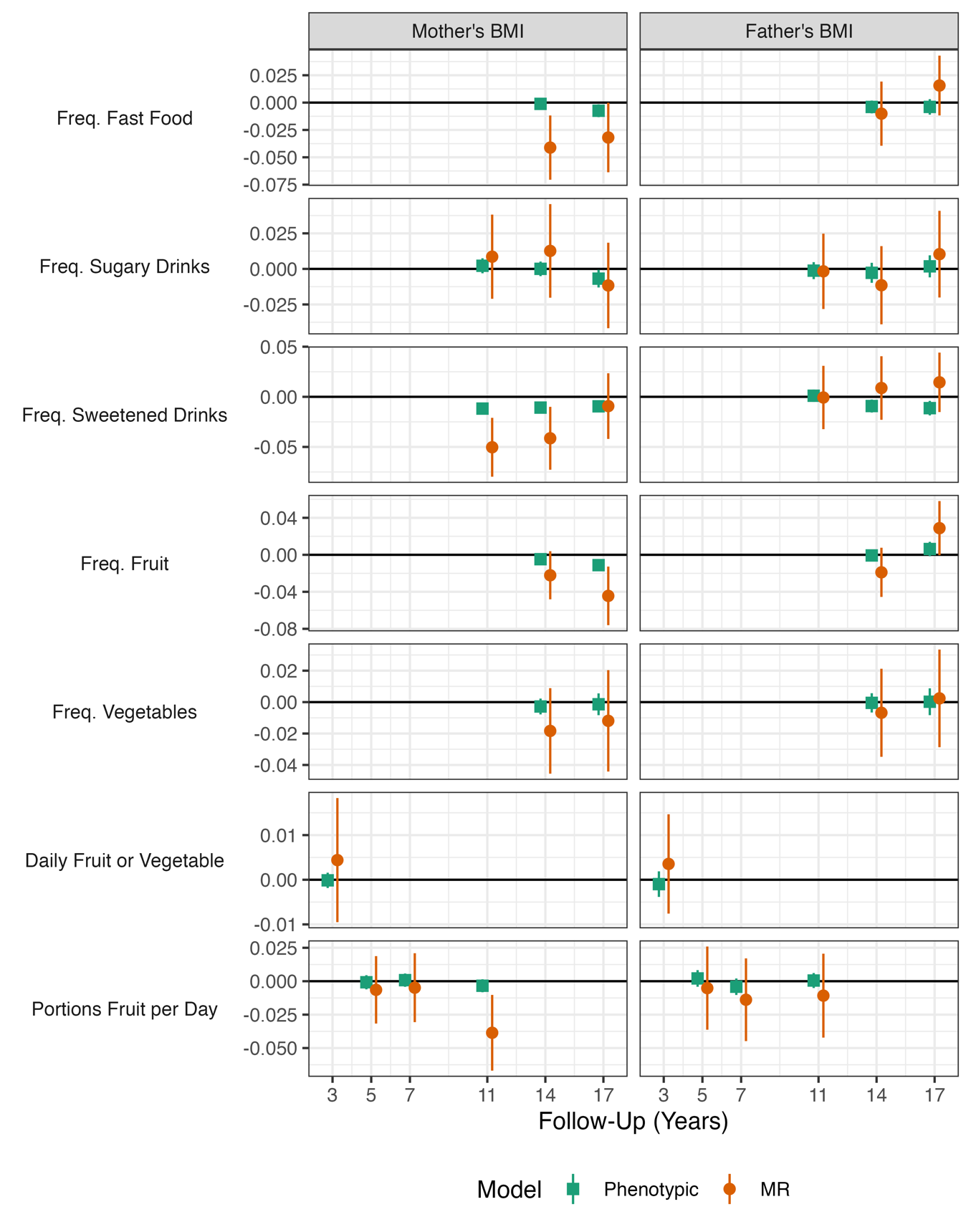


**Figure E: Association between mother’s and father’s BMI and offspring diet by survey sweep.** Results show the unit difference in the relevant offspring diet measure due to +1 kg/m^2^ increase in parental BMI. Derived from Mendelian Randomization (MR; IV 2SLS) and phenotypic multivariable regressions of offspring diet on mother’s and father’s BMI, with adjustment for sex, age at follow-up (two natural splines), maternal age at birth, family social class, mother’s education years, and 10 genetic principal components. In MR analysis, parental BMI instrumented with mother’s and father’s PGI. Diet variables are dichotomized (see Figure B for categorisation) and coded such that a value of 1 indicated a healthier diet (associations thus represent differences in probability). All regressions were weighted with non-response weights to account for selection into the genotyped study sample.


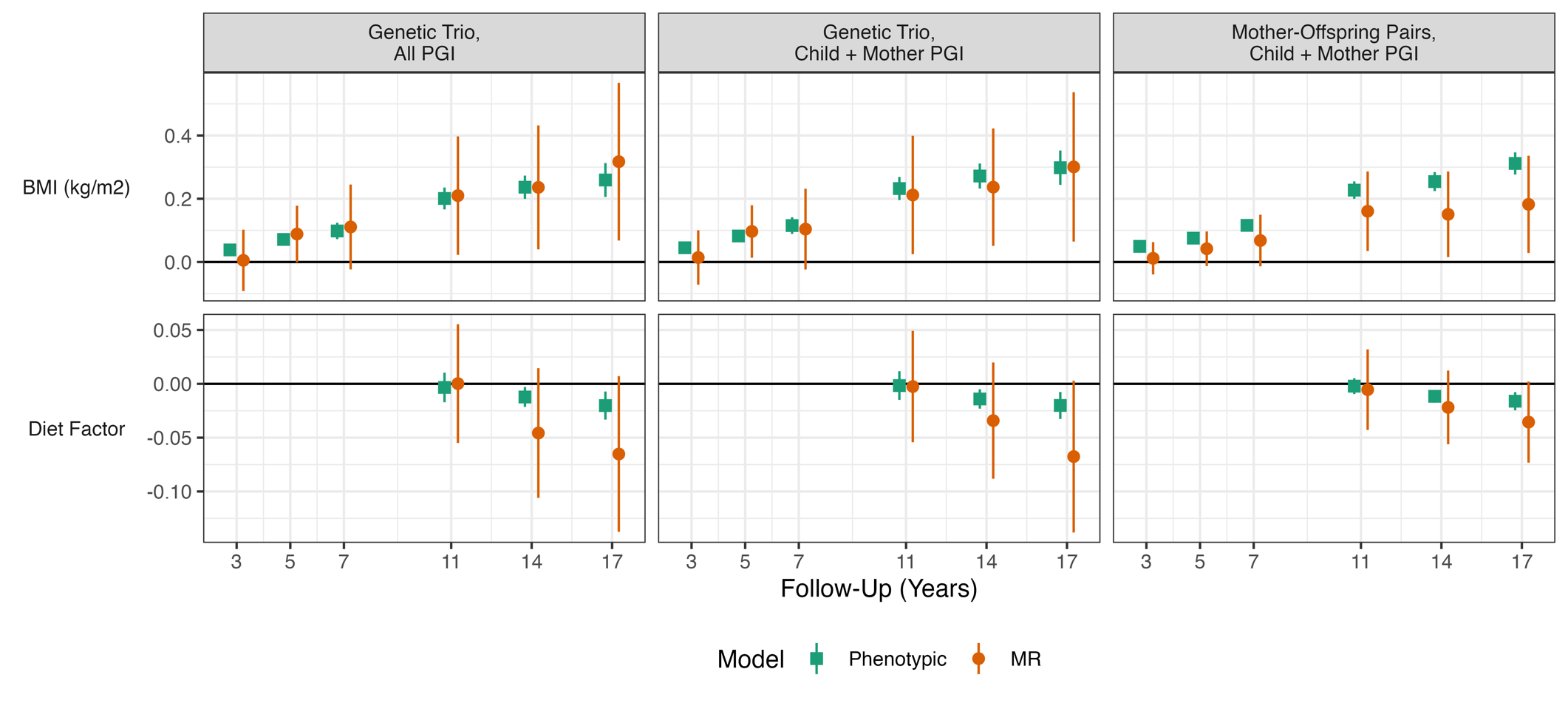


**Figure F: Association between mother’s BMI and offspring BMI and diet by sample, PGIs used, and survey sweep.** Results show the kg/m^2^ (BMI) or SD (diet) difference due to +1 kg/m^2^ increase in parental BMI. Derived from Mendelian Randomization (MR; IV 2SLS) and ‘phenotypic’ multivariable regressions of offspring adiposity on mother’s BMI, with adjustment for sex, age at follow-up (two natural splines), maternal age at birth, family social class, mother’s education years, and 10 genetic principal components. In MR analysis, mother’s BMI instrumented with mother’s PGI and additionally adjusted for offspring PGI. ‘Genetic Trio, All PGI’ refers to models using genetic trio sample and including father’s BMI as a covariate and, in MR analysis, father’s PGI as an instrument for this. ‘Genetic Trio, Child + Mother PGI’ refers to models of the same sample except father’s BMI and father’s PGI was not included. ‘Mother-Offspring Pairs, Child + Mother PGI’ refers to models using the mother-offspring pair genotyped sample, with father’s PGI or BMI again not included in as covariates or instruments. This regression doubled the sample size. All models also included adjustment for sex, age (two natural splines), maternal age at birth, family socioeconomic class, mother’s education years, and 10 genetic principal components. All regressions were weighted with non-response weights to account for selection into the genotyped study sample.


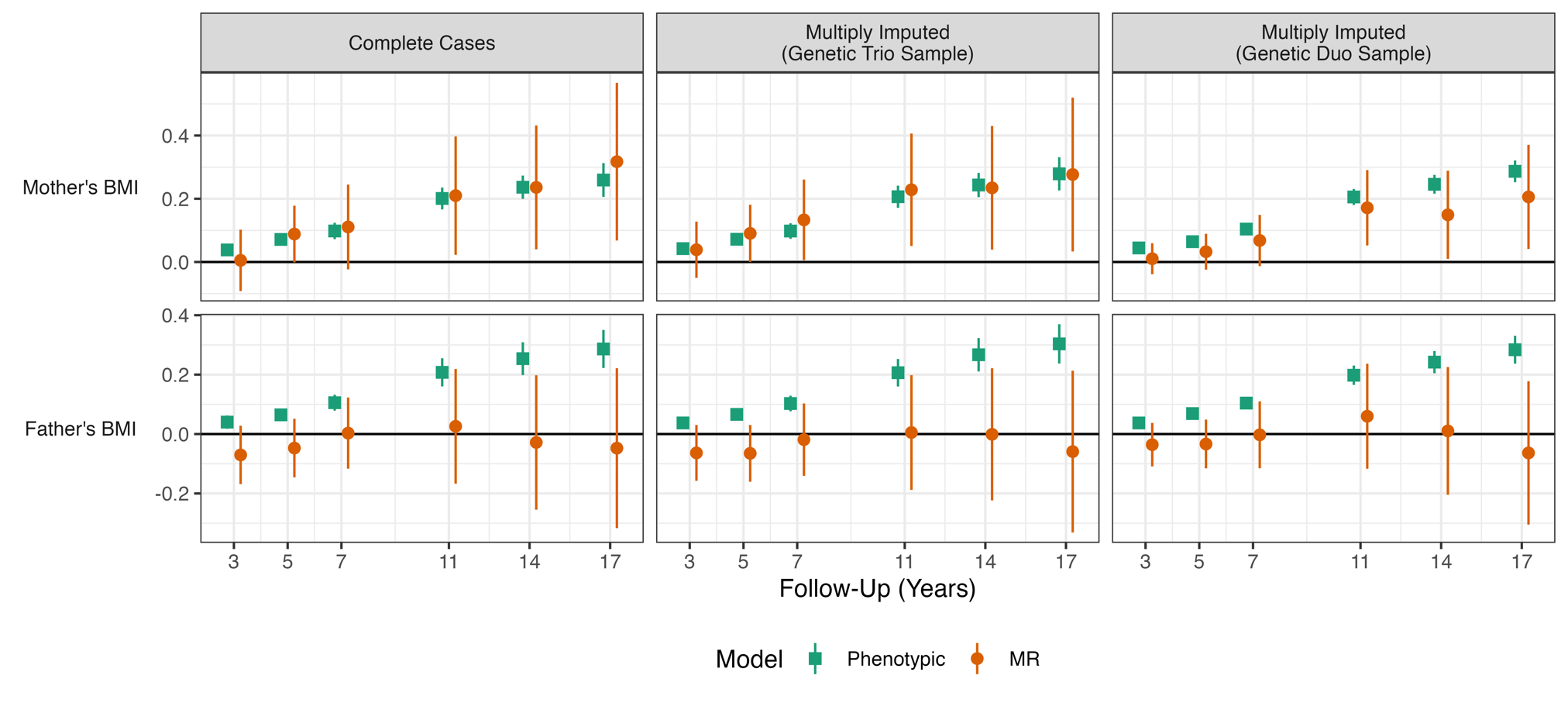


**Figure G: Association between mother’s and father’s BMI and offspring BMI (z-scores) by sample, PGIs used, and survey sweep.** Results show the kg/m^2^ difference in offspring BMI due to +1 kg/m^2^ increase in the relevant parent’s BMI. Derived from Mendelian Randomization (MR; IV 2SLS) and ‘phenotypic’ multivariable regressions of offspring BMI (z-score) on mother’s BMI, with adjustment for sex, age at follow-up (two natural splines), maternal age at birth, family social class, mother’s education years, and 10 genetic principal components. In MR analysis, parental BMI instrumented with mother’s and father’s PGI and additionally adjusted for offspring PGI. ‘Complete Cases’ refers to models using (outcome-sweep specific) complete cases data. ‘Multiply Imputed (Genetic Trio Sample)’ refers to models using multiply imputed data for the genotyped trio (mother-father-offspring) sample. ‘Multiply Imputed (Genetic Duo Sample)’ refers to models using multiply imputed data for the genotyped duo (mother-offspring or father-offspring) sample. All models also included adjustment for sex, age (two natural splines), maternal age at birth, family socioeconomic class, mother’s education years, and 10 genetic principal components. All regressions were weighted with non-response weights to account for selection into the genotyped study sample. Data were imputed with chained equations (40 imputations) in wide format, with covariates, PGIs, and BMI (z-scores) and diet MCA factor values included in imputation models. Estimates using multiply imputed data were pooled using Rubin’s (1987) rules.


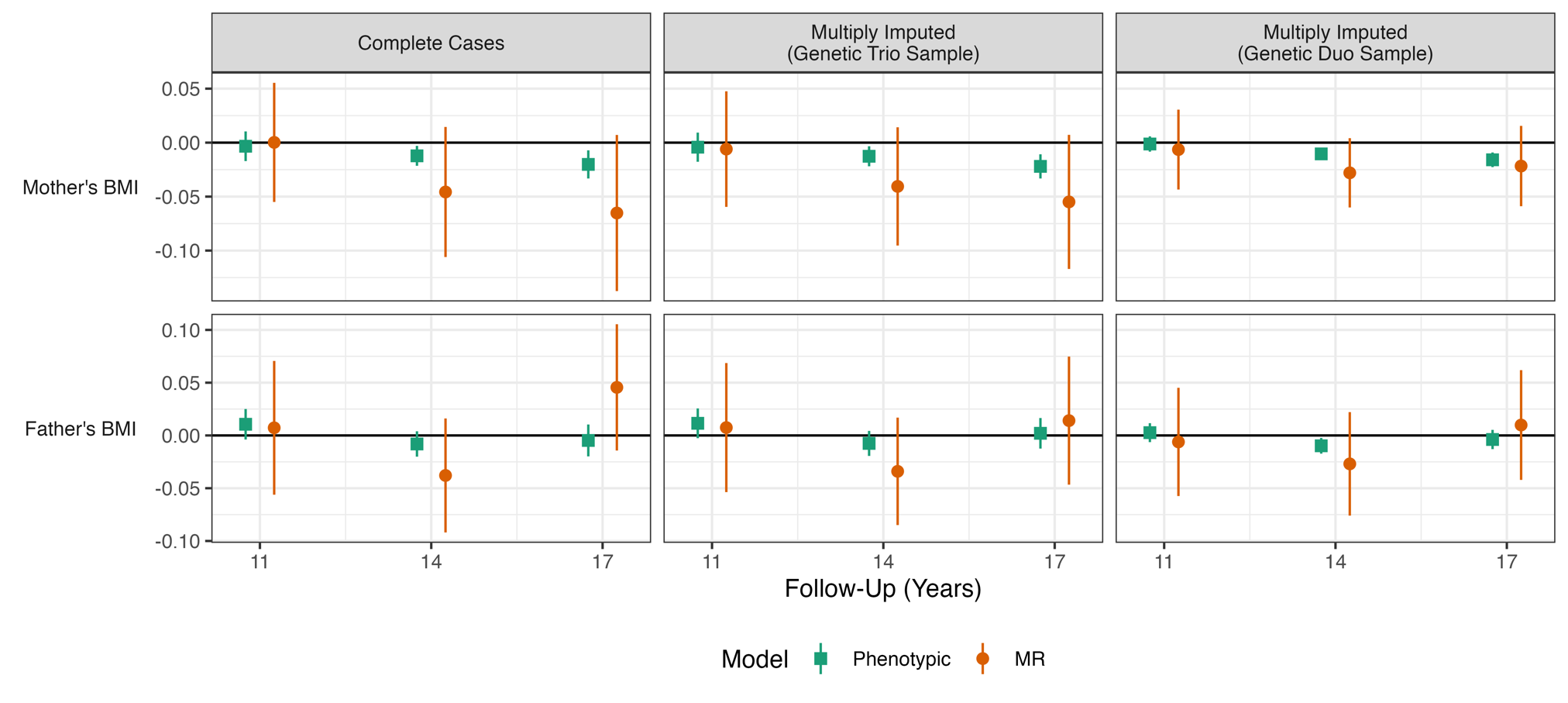


**Figure H: Association between mother’s and father’s BMI and offspring diet by sample, PGIs used, and survey sweep.** Results show the SD difference in offspring diet factor score due to +1 kg/m^2^ increase in the relevant parent’s BMI. Derived from Mendelian Randomization (MR; IV 2SLS) and ‘phenotypic’ multivariable regressions of offspring diet (MCA factor) on mother’s BMI, with adjustment for sex, age at follow-up (two natural splines), maternal age at birth, family social class, mother’s education years, and 10 genetic principal components. In MR analysis, parental BMI instrumented with mother’s and father’s PGI and additionally adjusted for offspring PGI. ‘Complete Cases’ refers to models using (outcome-sweep specific) complete cases data. ‘Multiply Imputed (Genetic Trio Sample)’ refers to models using multiply imputed data for the genotyped trio (mother-father-offspring) sample. ‘Multiply Imputed (Genetic Dup Sample)’ refers to models using multiply imputed data for the genotyped duo (mother-offspring or father-offspring) sample. All models also included adjustment for sex, age (two natural splines), maternal age at birth, family socioeconomic class, mother’s education years, and 10 genetic principal components. All regressions were weighted with non-response weights to account for selection into the genotyped study sample. Data were imputed with chained equations (40 imputations) in wide format, with covariates, PGIs, and BMI (z-scores) and diet MCA factor values included in imputation models. Estimates using multiply imputed data were pooled using Rubin’s (1987) rules.


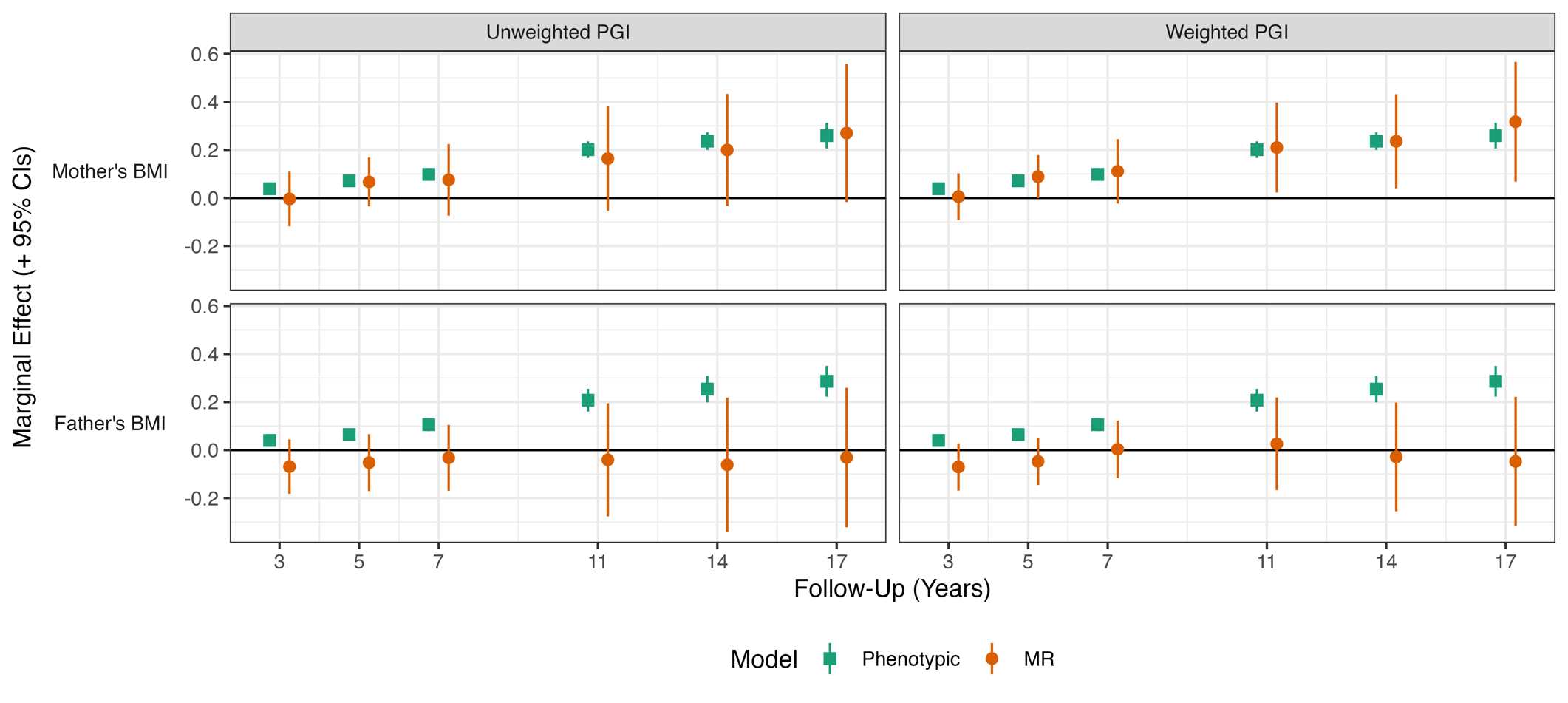


**Figure I: Association between mother’s and father’s BMI and offspring BMI by survey sweep and PGI used (weighted or unweighted).** Results show the kg/m^2^ difference in offspring BMI score due to +1 kg/m^2^ increase in the relevant parent’s BMI. Derived from Mendelian Randomization (MR; IV 2SLS) and (phenotypic) multivariable regression of BMI on mother’s and father’s BMI, with adjustment for sex, age at follow-up (two natural splines), maternal age at birth, family social class, mother’s education years, and 10 genetic principal components. In MR analysis, parental BMI instrumented with mother’s and father’s PGI with additional adjustment for offspring PGI. All regressions were weighted with non-response weights to account for selection into the genotyped study sample.


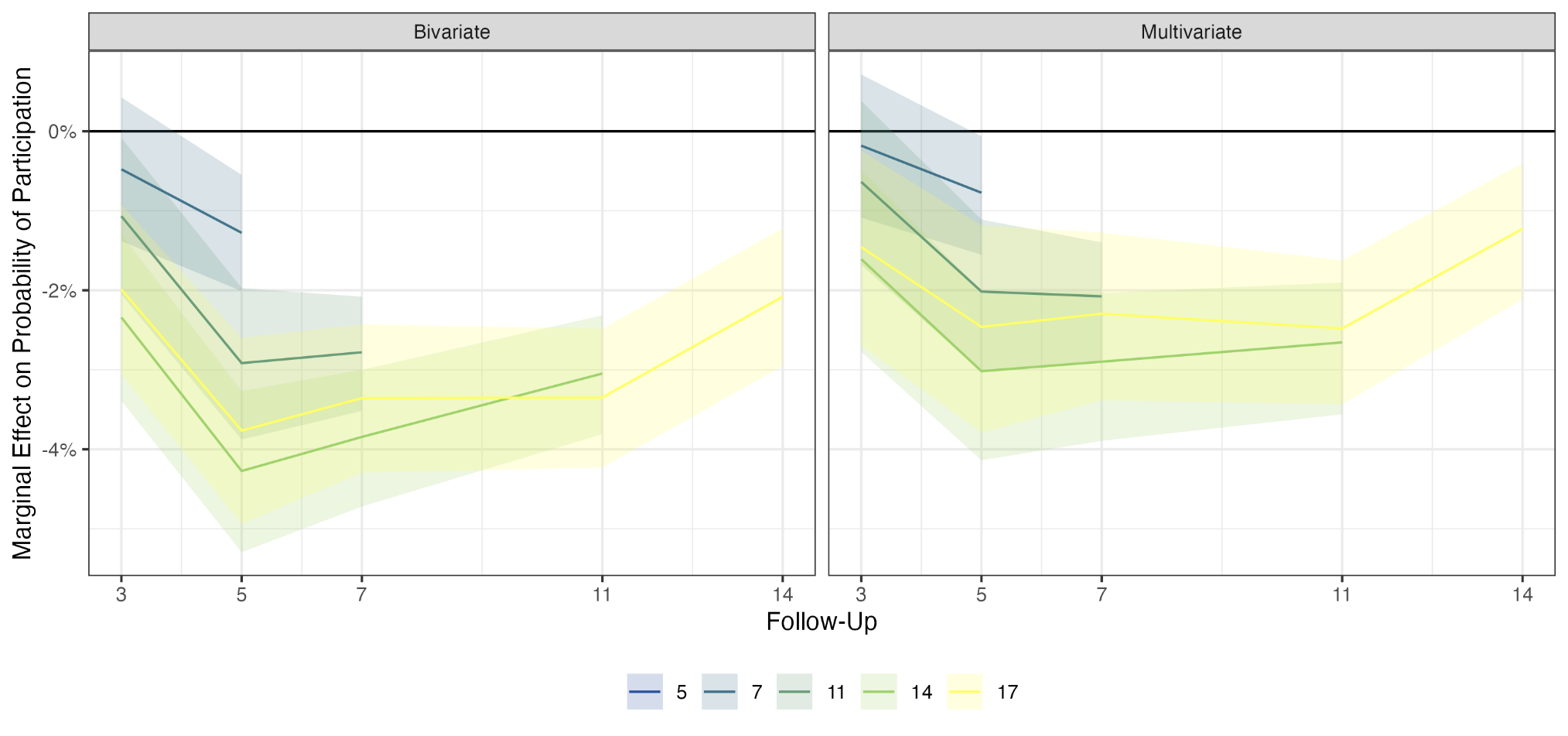


**Figure J: Difference in probability of participation in given survey sweep according to BMI (z-score) at prior sweep.** Derived from logistic regression of participation on BMI (z-score) from a specific sweep. Colour of line indicates participation sweep, while location on x-axis indicates sweep relevant BMI (z-scores) collected. Left panel shows univariate regression, while right panel includes adjustment for sex, family socioeconomic class, maternal age at birth, mother’s BMI, father’s BMI, and mother’s years of education. All regressions were weighted with non-response weights to account for selection into the genotyped study sample.

# Tables

**Table A: Regression Results, Phenotypic and Mendelian Randomization Models.** Association (+ 95% CI) between mother’s and father’s BMI and offspring diet and adiposity, by outcome variable, sweep, and estimator used. Genetic trio sample. ‘MR’ refers to Mendelian Randomization analysis using IV two stage least squares regression. In these models, parental BMI is instrumented with mother’s and father’s PGIs additionally adjusting for offspring PGI. ‘Phenotypic refers to multivariable regression analysis with parental (phenotypic) BMI entered into models directly (i.e. not instrumented with parental PGIs). ‘Z-Test’ refers to test examining differences in coefficients between MR and phenotypic models. Confidence intervals for this test were calculated using the Rao & Wu (1993) bootstrap method for clustered sampling designs (500 bootstraps, centile method; Kolenikov, 2010). All regressions were weighted with non-response weights to account for selection into the genotyped study sample and included adjustment for sex, age (two natural splines), maternal age at birth, family socioeconomic class, mother’s education and years 10 genetic principal components (PCs).

|  | | Mother's BMI | | | Father's BMI | | |
| --- | --- | --- | --- | --- | --- | --- | --- |
| Outcome | Follow-Up | Phenotypic | MR | Z-Test | Phenotypic | MR | Z-Test |
| BMI (kg/m2) | 3y | 0.04 (0.02, 0.06) | 0.01 (-0.09, 0.10) | -0.03 (-0.14, 0.05) | 0.04 (0.02, 0.06) | -0.07 (-0.17, 0.03) | -0.11 (-0.23, -0.02) |
|  | 5y | 0.07 (0.05, 0.09) | 0.09 (0.00, 0.18) | 0.01 (-0.08, 0.12) | 0.06 (0.05, 0.08) | -0.05 (-0.15, 0.05) | -0.11 (-0.23, 0.00) |
|  | 7y | 0.10 (0.07, 0.12) | 0.11 (-0.02, 0.24) | 0.01 (-0.12, 0.15) | 0.11 (0.08, 0.13) | 0.00 (-0.12, 0.12) | -0.10 (-0.23, 0.02) |
|  | 11y | 0.20 (0.17, 0.24) | 0.21 (0.02, 0.40) | 0.00 (-0.20, 0.19) | 0.21 (0.16, 0.26) | 0.03 (-0.17, 0.22) | -0.18 (-0.37, 0.04) |
|  | 14y | 0.24 (0.20, 0.27) | 0.24 (0.04, 0.43) | -0.01 (-0.23, 0.17) | 0.25 (0.20, 0.31) | -0.03 (-0.25, 0.20) | -0.27 (-0.54, -0.07) |
|  | 17y | 0.26 (0.21, 0.31) | 0.32 (0.07, 0.57) | 0.05 (-0.25, 0.29) | 0.29 (0.22, 0.35) | -0.05 (-0.32, 0.22) | -0.34 (-0.64, -0.07) |
| Birthweight (g) | 0y | 15.18 (9.61, 20.75) | 24.02 (-5.48, 53.52) | 7.98 (-25.90, 38.58) | 2.38 (-4.34, 9.11) | -16.18 (-44.81, 12.46) | -18.99 (-55.58, 7.93) |
| Height (m) | 3y | 0.00 (0.00, 0.00) | 0.00 (0.00, 0.00) | 0.00 (0.00, 0.00) | 0.00 (0.00, 0.00) | 0.00 (0.00, 0.00) | 0.00 (0.00, 0.00) |
|  | 5y | 0.00 (0.00, 0.00) | 0.00 (0.00, 0.00) | 0.00 (0.00, 0.00) | 0.00 (0.00, 0.00) | 0.00 (0.00, 0.00) | 0.00 (0.00, 0.00) |
|  | 7y | 0.00 (0.00, 0.00) | 0.00 (0.00, 0.01) | 0.00 (0.00, 0.01) | 0.00 (0.00, 0.00) | 0.00 (0.00, 0.00) | 0.00 (0.00, 0.00) |
|  | 11y | 0.00 (0.00, 0.00) | 0.00 (0.00, 0.01) | 0.00 (0.00, 0.01) | 0.00 (0.00, 0.00) | 0.00 (0.00, 0.01) | 0.00 (-0.01, 0.00) |
|  | 14y | 0.00 (0.00, 0.00) | 0.00 (0.00, 0.01) | 0.00 (0.00, 0.01) | 0.00 (0.00, 0.00) | 0.00 (-0.01, 0.00) | 0.00 (-0.01, 0.00) |
|  | 17y | 0.00 (0.00, 0.00) | 0.00 (0.00, 0.01) | 0.00 (0.00, 0.01) | 0.00 (0.00, 0.00) | 0.00 (0.00, 0.00) | 0.00 (-0.01, 0.00) |
| Weight (kg) | 3y | 0.03 (0.01, 0.06) | 0.04 (-0.07, 0.16) | 0.01 (-0.12, 0.14) | 0.06 (0.04, 0.09) | -0.04 (-0.17, 0.09) | -0.10 (-0.25, 0.02) |
|  | 5y | 0.10 (0.06, 0.13) | 0.17 (0.01, 0.32) | 0.07 (-0.10, 0.23) | 0.12 (0.09, 0.16) | -0.03 (-0.22, 0.15) | -0.16 (-0.36, 0.01) |
|  | 7y | 0.18 (0.12, 0.24) | 0.28 (0.03, 0.54) | 0.10 (-0.16, 0.35) | 0.22 (0.16, 0.28) | 0.02 (-0.24, 0.29) | -0.20 (-0.46, 0.06) |
|  | 11y | 0.48 (0.39, 0.58) | 0.54 (0.02, 1.07) | 0.05 (-0.50, 0.56) | 0.56 (0.44, 0.69) | 0.07 (-0.52, 0.65) | -0.50 (-1.10, 0.10) |
|  | 14y | 0.71 (0.59, 0.82) | 0.96 (0.28, 1.63) | 0.22 (-0.52, 0.94) | 0.76 (0.59, 0.93) | -0.21 (-1.01, 0.59) | -0.98 (-1.81, -0.16) |
|  | 17y | 0.71 (0.54, 0.89) | 1.07 (0.28, 1.87) | 0.33 (-0.51, 1.08) | 0.94 (0.75, 1.14) | -0.19 (-1.11, 0.74) | -1.13 (-2.09, -0.18) |
| BMI (Z-Score) | 3y | 0.03 (0.01, 0.04) | 0.01 (-0.06, 0.07) | -0.02 (-0.09, 0.04) | 0.02 (0.01, 0.04) | -0.05 (-0.11, 0.02) | -0.07 (-0.15, -0.01) |
|  | 5y | 0.04 (0.03, 0.06) | 0.06 (0.00, 0.12) | 0.01 (-0.04, 0.07) | 0.04 (0.03, 0.05) | -0.03 (-0.09, 0.03) | -0.07 (-0.14, 0.00) |
|  | 7y | 0.05 (0.04, 0.06) | 0.06 (0.00, 0.13) | 0.01 (-0.05, 0.08) | 0.05 (0.04, 0.06) | 0.01 (-0.05, 0.07) | -0.04 (-0.10, 0.02) |
|  | 11y | 0.07 (0.06, 0.08) | 0.08 (0.01, 0.14) | 0.01 (-0.07, 0.08) | 0.07 (0.06, 0.09) | 0.01 (-0.06, 0.08) | -0.06 (-0.13, 0.02) |
|  | 14y | 0.07 (0.06, 0.08) | 0.08 (0.01, 0.14) | 0.00 (-0.06, 0.06) | 0.08 (0.06, 0.09) | -0.01 (-0.08, 0.06) | -0.09 (-0.16, -0.02) |
|  | 17y | 0.07 (0.06, 0.08) | 0.10 (0.02, 0.17) | 0.02 (-0.06, 0.10) | 0.09 (0.07, 0.10) | -0.01 (-0.08, 0.07) | -0.09 (-0.17, -0.02) |
| Weight (Z-Score) | 3y | 0.02 (0.00, 0.03) | 0.02 (-0.04, 0.09) | 0.01 (-0.06, 0.08) | 0.03 (0.02, 0.05) | -0.03 (-0.10, 0.05) | -0.06 (-0.14, 0.01) |
|  | 5y | 0.03 (0.02, 0.05) | 0.07 (0.01, 0.13) | 0.03 (-0.03, 0.09) | 0.04 (0.03, 0.05) | -0.01 (-0.08, 0.05) | -0.06 (-0.13, 0.00) |
|  | 7y | 0.04 (0.03, 0.06) | 0.07 (0.01, 0.14) | 0.03 (-0.03, 0.09) | 0.05 (0.04, 0.06) | 0.01 (-0.05, 0.08) | -0.04 (-0.10, 0.03) |
|  | 11y | 0.06 (0.04, 0.07) | 0.07 (0.01, 0.13) | 0.01 (-0.05, 0.07) | 0.06 (0.05, 0.08) | 0.00 (-0.07, 0.07) | -0.06 (-0.13, 0.01) |
|  | 14y | 0.06 (0.05, 0.07) | 0.09 (0.03, 0.15) | 0.02 (-0.05, 0.09) | 0.07 (0.06, 0.09) | -0.02 (-0.09, 0.06) | -0.09 (-0.17, -0.01) |
|  | 17y | 0.06 (0.05, 0.08) | 0.10 (0.03, 0.18) | 0.03 (-0.04, 0.11) | 0.09 (0.07, 0.10) | -0.01 (-0.10, 0.07) | -0.10 (-0.19, -0.01) |
| Height (Z-Score) | 3y | 0.00 (-0.01, 0.01) | 0.02 (-0.03, 0.08) | 0.02 (-0.03, 0.09) | 0.02 (0.01, 0.04) | -0.01 (-0.07, 0.06) | -0.03 (-0.10, 0.03) |
|  | 5y | 0.00 (-0.01, 0.02) | 0.02 (-0.03, 0.08) | 0.02 (-0.04, 0.08) | 0.03 (0.01, 0.04) | 0.01 (-0.05, 0.07) | -0.01 (-0.08, 0.05) |
|  | 7y | 0.01 (0.00, 0.02) | 0.04 (-0.02, 0.10) | 0.03 (-0.02, 0.10) | 0.02 (0.01, 0.04) | 0.00 (-0.07, 0.06) | -0.03 (-0.10, 0.04) |
|  | 11y | 0.01 (0.00, 0.03) | 0.04 (-0.03, 0.10) | 0.02 (-0.04, 0.09) | 0.03 (0.01, 0.04) | 0.01 (-0.06, 0.07) | -0.02 (-0.10, 0.04) |
|  | 14y | 0.01 (0.00, 0.03) | 0.05 (-0.01, 0.11) | 0.04 (-0.03, 0.10) | 0.01 (0.00, 0.03) | -0.03 (-0.09, 0.04) | -0.04 (-0.11, 0.02) |
|  | 17y | -0.01 (-0.02, 0.00) | 0.02 (-0.04, 0.08) | 0.02 (-0.03, 0.09) | 0.02 (0.00, 0.03) | 0.00 (-0.07, 0.06) | -0.02 (-0.09, 0.05) |
| Body Fat (%) | 7y | 0.19 (0.13, 0.24) | 0.20 (-0.07, 0.46) | -0.01 (-0.29, 0.27) | 0.18 (0.12, 0.25) | -0.01 (-0.27, 0.24) | -0.19 (-0.48, 0.05) |
|  | 11y | 0.39 (0.32, 0.46) | 0.29 (-0.11, 0.69) | -0.09 (-0.53, 0.30) | 0.39 (0.30, 0.49) | 0.27 (-0.10, 0.65) | -0.12 (-0.50, 0.34) |
|  | 14y | 0.41 (0.33, 0.49) | 0.35 (-0.01, 0.72) | -0.09 (-0.49, 0.26) | 0.46 (0.35, 0.56) | 0.09 (-0.33, 0.50) | -0.38 (-0.81, 0.03) |
|  | 17y | 0.42 (0.32, 0.52) | 0.25 (-0.20, 0.70) | -0.18 (-0.62, 0.22) | 0.44 (0.33, 0.55) | -0.08 (-0.52, 0.36) | -0.52 (-0.99, 0.00) |
| Fat-to-Non-Fat Ratio | 7y | 0.00 (0.00, 0.00) | 0.00 (0.00, 0.01) | 0.00 (-0.01, 0.00) | 0.00 (0.00, 0.00) | 0.00 (0.00, 0.00) | 0.00 (-0.01, 0.00) |
|  | 11y | 0.01 (0.01, 0.01) | 0.00 (0.00, 0.01) | 0.00 (-0.01, 0.01) | 0.01 (0.01, 0.01) | 0.00 (0.00, 0.01) | 0.00 (-0.01, 0.01) |
|  | 14y | 0.01 (0.01, 0.01) | 0.01 (0.00, 0.01) | 0.00 (-0.01, 0.00) | 0.01 (0.01, 0.01) | 0.00 (-0.01, 0.01) | -0.01 (-0.01, 0.00) |
|  | 17y | 0.01 (0.01, 0.01) | 0.00 (0.00, 0.01) | 0.00 (-0.01, 0.00) | 0.01 (0.01, 0.01) | 0.00 (-0.01, 0.01) | -0.01 (-0.02, 0.00) |
| Fat Mass Index | 7y | 0.03 (0.02, 0.04) | 0.03 (-0.02, 0.07) | 0.00 (-0.05, 0.04) | 0.03 (0.02, 0.03) | 0.01 (-0.03, 0.06) | -0.01 (-0.05, 0.03) |
|  | 11y | 0.04 (0.03, 0.05) | 0.03 (-0.01, 0.07) | -0.01 (-0.06, 0.03) | 0.03 (0.02, 0.04) | 0.01 (-0.02, 0.05) | -0.02 (-0.06, 0.02) |
|  | 14y | 0.09 (0.07, 0.10) | 0.08 (0.00, 0.15) | -0.01 (-0.09, 0.07) | 0.09 (0.07, 0.11) | 0.02 (-0.07, 0.11) | -0.07 (-0.16, 0.01) |
|  | 17y | 0.10 (0.07, 0.13) | 0.15 (0.03, 0.27) | 0.04 (-0.09, 0.15) | 0.11 (0.08, 0.14) | -0.01 (-0.13, 0.12) | -0.12 (-0.24, 0.02) |
| Fat-Free Mass Index | 7y | 0.03 (0.02, 0.04) | 0.05 (0.00, 0.10) | 0.02 (-0.03, 0.07) | 0.04 (0.03, 0.05) | 0.03 (-0.02, 0.08) | -0.01 (-0.05, 0.05) |
|  | 11y | 0.05 (0.04, 0.06) | 0.06 (0.00, 0.12) | 0.01 (-0.05, 0.08) | 0.05 (0.04, 0.06) | -0.01 (-0.07, 0.05) | -0.06 (-0.12, 0.00) |
|  | 14y | 0.06 (0.05, 0.08) | 0.09 (0.03, 0.16) | 0.03 (-0.04, 0.09) | 0.08 (0.06, 0.10) | 0.00 (-0.07, 0.07) | -0.08 (-0.15, -0.02) |
|  | 17y | 0.09 (0.07, 0.12) | 0.20 (0.09, 0.32) | 0.11 (-0.02, 0.23) | 0.13 (0.10, 0.16) | 0.00 (-0.12, 0.13) | -0.12 (-0.26, 0.02) |
| Waist-to-Height Ratio | 5y | 0.00 (0.00, 0.00) | 0.00 (0.00, 0.00) | 0.00 (0.00, 0.00) | 0.00 (0.00, 0.00) | 0.00 (0.00, 0.00) | 0.00 (0.00, 0.00) |
|  | 7y | 0.00 (0.00, 0.00) | 0.00 (0.00, 0.00) | 0.00 (0.00, 0.00) | 0.00 (0.00, 0.00) | 0.00 (0.00, 0.00) | 0.00 (0.00, 0.00) |
| Diet Factor | 11y | 0.00 (-0.02, 0.01) | 0.00 (-0.05, 0.06) | 0.00 (-0.06, 0.06) | 0.01 (0.00, 0.03) | 0.01 (-0.06, 0.07) | 0.00 (-0.07, 0.06) |
|  | 14y | -0.01 (-0.02, 0.00) | -0.05 (-0.11, 0.01) | -0.04 (-0.10, 0.03) | -0.01 (-0.02, 0.00) | -0.04 (-0.09, 0.02) | -0.03 (-0.10, 0.02) |
|  | 17y | -0.02 (-0.03, -0.01) | -0.07 (-0.14, 0.01) | -0.04 (-0.12, 0.02) | 0.00 (-0.02, 0.01) | 0.05 (-0.01, 0.11) | 0.05 (-0.02, 0.12) |
| Freq. Fast Food | 14y | 0.00 (-0.01, 0.00) | -0.04 (-0.07, -0.01) | -0.04 (-0.07, -0.01) | 0.00 (-0.01, 0.00) | -0.01 (-0.04, 0.02) | 0.00 (-0.04, 0.02) |
|  | 17y | -0.01 (-0.01, 0.00) | -0.03 (-0.06, 0.00) | -0.02 (-0.06, 0.01) | 0.00 (-0.01, 0.00) | 0.02 (-0.01, 0.04) | 0.02 (-0.01, 0.05) |
| Freq. Sugary Drinks | 11y | 0.00 (0.00, 0.01) | 0.01 (-0.02, 0.04) | 0.01 (-0.02, 0.04) | 0.00 (-0.01, 0.00) | 0.00 (-0.03, 0.02) | 0.00 (-0.03, 0.03) |
|  | 14y | 0.00 (-0.01, 0.01) | 0.01 (-0.02, 0.05) | 0.01 (-0.02, 0.05) | 0.00 (-0.01, 0.00) | -0.01 (-0.04, 0.02) | -0.01 (-0.03, 0.02) |
|  | 17y | -0.01 (-0.01, 0.00) | -0.01 (-0.04, 0.02) | 0.00 (-0.04, 0.03) | 0.00 (-0.01, 0.01) | 0.01 (-0.02, 0.04) | 0.01 (-0.02, 0.04) |
| Freq. Sweetened Drinks | 11y | -0.01 (-0.02, -0.01) | -0.05 (-0.08, -0.02) | -0.04 (-0.08, -0.01) | 0.00 (-0.01, 0.01) | 0.00 (-0.03, 0.03) | 0.00 (-0.04, 0.03) |
|  | 14y | -0.01 (-0.02, -0.01) | -0.04 (-0.07, -0.01) | -0.03 (-0.07, 0.00) | -0.01 (-0.02, 0.00) | 0.01 (-0.02, 0.04) | 0.02 (-0.02, 0.05) |
|  | 17y | -0.01 (-0.02, 0.00) | -0.01 (-0.04, 0.02) | 0.00 (-0.03, 0.03) | -0.01 (-0.02, 0.00) | 0.01 (-0.02, 0.04) | 0.03 (0.00, 0.06) |
| Freq. Fruit | 14y | 0.00 (-0.01, 0.00) | -0.02 (-0.05, 0.00) | -0.02 (-0.04, 0.01) | 0.00 (-0.01, 0.00) | -0.02 (-0.05, 0.01) | -0.02 (-0.05, 0.01) |
|  | 17y | -0.01 (-0.02, -0.01) | -0.04 (-0.08, -0.01) | -0.03 (-0.07, 0.00) | 0.01 (0.00, 0.01) | 0.03 (0.00, 0.06) | 0.02 (-0.01, 0.06) |
| Freq. Vegetables | 14y | 0.00 (-0.01, 0.00) | -0.02 (-0.05, 0.01) | -0.02 (-0.05, 0.01) | 0.00 (-0.01, 0.01) | -0.01 (-0.03, 0.02) | -0.01 (-0.04, 0.02) |
|  | 17y | 0.00 (-0.01, 0.01) | -0.01 (-0.04, 0.02) | -0.01 (-0.04, 0.02) | 0.00 (-0.01, 0.01) | 0.00 (-0.03, 0.03) | 0.00 (-0.03, 0.03) |
| Daily Fruit or Vegetable | 3y | 0.00 (0.00, 0.00) | 0.00 (-0.01, 0.02) | 0.00 (-0.01, 0.02) | 0.00 (0.00, 0.00) | 0.00 (-0.01, 0.01) | 0.00 (-0.01, 0.02) |
| Portions Fruit per Day | 5y | 0.00 (-0.01, 0.00) | -0.01 (-0.03, 0.02) | -0.01 (-0.04, 0.02) | 0.00 (0.00, 0.01) | -0.01 (-0.04, 0.03) | -0.01 (-0.04, 0.03) |
|  | 7y | 0.00 (0.00, 0.01) | 0.00 (-0.03, 0.02) | -0.01 (-0.04, 0.02) | 0.00 (-0.01, 0.00) | -0.01 (-0.04, 0.02) | -0.01 (-0.05, 0.02) |
|  | 11y | 0.00 (-0.01, 0.00) | -0.04 (-0.07, -0.01) | -0.04 (-0.07, 0.00) | 0.00 (-0.01, 0.01) | -0.01 (-0.04, 0.02) | -0.01 (-0.05, 0.02) |

**Table B: Regression Results, Association Between PGI and Offspring Phenotypes.** Association (+ 95% CI) between child’s, father’s, or mother’s PGI and offspring BMI (z-score) or diet, by sweep. Genetic trio sample. Associations calculated using OLS regression. Child’s, father’s and mother’s PGIs entered into the models simultaneously. Child’s PGI reflects direct genetic effects and father’s and mother’s PGIs reflect indirect genetic effects. Regressions were weighted with non-response weights to account for selection into the genotyped study sample and included adjustment for sex, age (two natural splines), maternal age at birth, mother’s education years, and 10 genetic principal components (PCs).

|  | | PGI | | |
| --- | --- | --- | --- | --- |
| Outcome | Follow-Up | Child's | Mother's | Father's |
| BMI (kg/m2) | 3y | 0.14 (0.04, 0.25) | -0.01 (-0.10, 0.09) | -0.07 (-0.16, 0.01) |
|  | 5y | 0.19 (0.08, 0.29) | 0.09 (-0.01, 0.18) | -0.05 (-0.14, 0.04) |
|  | 7y | 0.31 (0.16, 0.47) | 0.11 (-0.03, 0.25) | 0.00 (-0.11, 0.12) |
|  | 11y | 0.64 (0.43, 0.85) | 0.22 (0.02, 0.42) | 0.02 (-0.17, 0.20) |
|  | 14y | 0.81 (0.55, 1.06) | 0.24 (0.03, 0.44) | -0.02 (-0.23, 0.19) |
|  | 17y | 1.06 (0.73, 1.39) | 0.29 (0.02, 0.57) | -0.05 (-0.31, 0.21) |
| Birthweight (g) | 0y | 14.71 (-17.86, 47.28) | 20.11 (-8.73, 48.95) | -13.27 (-39.42, 12.87) |
| Height (m) | 3y | 0.00 (0.00, 0.00) | 0.00 (0.00, 0.00) | 0.00 (0.00, 0.00) |
|  | 5y | 0.00 (0.00, 0.00) | 0.00 (0.00, 0.00) | 0.00 (0.00, 0.00) |
|  | 7y | 0.00 (0.00, 0.00) | 0.00 (0.00, 0.01) | 0.00 (0.00, 0.00) |
|  | 11y | 0.00 (0.00, 0.01) | 0.00 (0.00, 0.01) | 0.00 (0.00, 0.00) |
|  | 14y | 0.00 (0.00, 0.01) | 0.00 (0.00, 0.01) | 0.00 (-0.01, 0.00) |
|  | 17y | 0.00 (-0.01, 0.00) | 0.00 (0.00, 0.01) | 0.00 (0.00, 0.00) |
| Weight (kg) | 3y | 0.10 (-0.03, 0.24) | 0.03 (-0.09, 0.15) | -0.05 (-0.17, 0.07) |
|  | 5y | 0.25 (0.07, 0.44) | 0.17 (0.00, 0.33) | -0.04 (-0.21, 0.14) |
|  | 7y | 0.49 (0.18, 0.80) | 0.28 (0.02, 0.55) | 0.02 (-0.24, 0.28) |
|  | 11y | 1.54 (0.96, 2.13) | 0.56 (0.00, 1.11) | 0.01 (-0.55, 0.58) |
|  | 14y | 2.23 (1.36, 3.09) | 0.92 (0.23, 1.61) | -0.18 (-0.90, 0.55) |
|  | 17y | 2.88 (1.82, 3.93) | 1.01 (0.15, 1.87) | -0.20 (-1.06, 0.65) |
| BMI (Z-Score) | 3y | 0.10 (0.03, 0.17) | 0.00 (-0.06, 0.06) | -0.05 (-0.11, 0.01) |
|  | 5y | 0.12 (0.05, 0.18) | 0.06 (0.00, 0.12) | -0.03 (-0.09, 0.02) |
|  | 7y | 0.16 (0.08, 0.23) | 0.07 (0.00, 0.13) | 0.01 (-0.04, 0.06) |
|  | 11y | 0.24 (0.16, 0.31) | 0.08 (0.01, 0.15) | 0.01 (-0.05, 0.07) |
|  | 14y | 0.27 (0.19, 0.34) | 0.08 (0.01, 0.14) | -0.01 (-0.07, 0.05) |
|  | 17y | 0.31 (0.21, 0.40) | 0.09 (0.01, 0.17) | 0.00 (-0.07, 0.07) |
| Weight (Z-Score) | 3y | 0.06 (-0.01, 0.14) | 0.02 (-0.05, 0.08) | -0.03 (-0.09, 0.04) |
|  | 5y | 0.09 (0.02, 0.16) | 0.07 (0.00, 0.13) | -0.01 (-0.08, 0.05) |
|  | 7y | 0.11 (0.04, 0.19) | 0.08 (0.01, 0.14) | 0.01 (-0.05, 0.07) |
|  | 11y | 0.19 (0.12, 0.25) | 0.07 (0.00, 0.13) | 0.00 (-0.06, 0.06) |
|  | 14y | 0.21 (0.13, 0.29) | 0.09 (0.02, 0.15) | -0.01 (-0.08, 0.05) |
|  | 17y | 0.26 (0.16, 0.36) | 0.09 (0.01, 0.18) | -0.01 (-0.09, 0.07) |
| Height (Z-Score) | 3y | 0.00 (-0.07, 0.07) | 0.02 (-0.04, 0.08) | -0.01 (-0.07, 0.05) |
|  | 5y | 0.03 (-0.03, 0.10) | 0.02 (-0.04, 0.08) | 0.01 (-0.05, 0.07) |
|  | 7y | 0.02 (-0.05, 0.09) | 0.04 (-0.03, 0.10) | 0.00 (-0.07, 0.06) |
|  | 11y | 0.04 (-0.04, 0.11) | 0.03 (-0.03, 0.10) | 0.00 (-0.06, 0.06) |
|  | 14y | 0.02 (-0.05, 0.09) | 0.04 (-0.02, 0.10) | -0.02 (-0.08, 0.04) |
|  | 17y | -0.03 (-0.10, 0.05) | 0.02 (-0.04, 0.08) | -0.01 (-0.07, 0.06) |
| Body Fat (%) | 7y | 0.59 (0.27, 0.91) | 0.21 (-0.07, 0.49) | 0.00 (-0.26, 0.26) |
|  | 11y | 1.08 (0.65, 1.51) | 0.38 (-0.03, 0.78) | 0.27 (-0.11, 0.64) |
|  | 14y | 1.29 (0.80, 1.77) | 0.36 (-0.02, 0.75) | 0.09 (-0.31, 0.49) |
|  | 17y | 1.63 (1.08, 2.17) | 0.21 (-0.27, 0.69) | -0.04 (-0.47, 0.39) |
| Fat-to-Non-Fat Ratio | 7y | 0.01 (0.00, 0.01) | 0.00 (0.00, 0.01) | 0.00 (0.00, 0.00) |
|  | 11y | 0.02 (0.01, 0.02) | 0.01 (0.00, 0.01) | 0.01 (0.00, 0.01) |
|  | 14y | 0.02 (0.01, 0.03) | 0.01 (0.00, 0.01) | 0.00 (-0.01, 0.01) |
|  | 17y | 0.03 (0.02, 0.04) | 0.00 (-0.01, 0.01) | 0.00 (-0.01, 0.01) |
| Fat Mass Index | 7y | 0.07 (0.01, 0.12) | 0.03 (-0.02, 0.08) | 0.02 (-0.02, 0.06) |
|  | 11y | 0.12 (0.08, 0.16) | 0.03 (-0.01, 0.07) | 0.02 (-0.02, 0.05) |
|  | 14y | 0.28 (0.18, 0.38) | 0.08 (0.00, 0.16) | 0.02 (-0.06, 0.10) |
|  | 17y | 0.38 (0.24, 0.52) | 0.14 (0.01, 0.27) | 0.01 (-0.10, 0.12) |
| Fat-Free Mass Index | 7y | 0.09 (0.03, 0.14) | 0.06 (0.00, 0.11) | 0.03 (-0.01, 0.07) |
|  | 11y | 0.18 (0.12, 0.25) | 0.06 (0.00, 0.12) | -0.01 (-0.06, 0.05) |
|  | 14y | 0.25 (0.17, 0.33) | 0.09 (0.03, 0.16) | 0.00 (-0.06, 0.07) |
|  | 17y | 0.38 (0.23, 0.52) | 0.20 (0.08, 0.32) | 0.02 (-0.09, 0.13) |
| Waist-to-Height Ratio | 5y | 0.00 (0.00, 0.01) | 0.00 (0.00, 0.00) | 0.00 (0.00, 0.00) |
|  | 7y | 0.00 (0.00, 0.01) | 0.00 (0.00, 0.00) | 0.00 (0.00, 0.00) |
| Diet Factor | 11y | 0.01 (-0.06, 0.07) | 0.00 (-0.06, 0.06) | 0.01 (-0.05, 0.07) |
|  | 14y | 0.06 (-0.01, 0.12) | -0.04 (-0.10, 0.01) | -0.03 (-0.08, 0.02) |
|  | 17y | -0.04 (-0.12, 0.04) | -0.06 (-0.13, 0.02) | 0.03 (-0.03, 0.09) |
| Freq. Fast Food | 14y | 0.05 (0.02, 0.08) | -0.04 (-0.07, -0.01) | -0.01 (-0.03, 0.01) |
|  | 17y | 0.02 (-0.02, 0.05) | -0.04 (-0.07, 0.00) | 0.01 (-0.03, 0.04) |
| Freq. Sugary Drinks | 11y | 0.00 (-0.04, 0.03) | 0.01 (-0.02, 0.04) | 0.00 (-0.03, 0.03) |
|  | 14y | 0.01 (-0.03, 0.04) | 0.01 (-0.02, 0.04) | -0.01 (-0.04, 0.01) |
|  | 17y | -0.01 (-0.05, 0.03) | -0.01 (-0.04, 0.02) | 0.01 (-0.02, 0.04) |
| Freq. Sweetened Drinks | 11y | 0.04 (0.00, 0.07) | -0.05 (-0.08, -0.02) | 0.00 (-0.03, 0.03) |
|  | 14y | -0.01 (-0.04, 0.02) | -0.04 (-0.07, -0.01) | 0.01 (-0.02, 0.03) |
|  | 17y | -0.03 (-0.07, 0.01) | -0.01 (-0.05, 0.02) | 0.01 (-0.02, 0.04) |
| Freq. Fruit | 14y | 0.04 (0.01, 0.06) | -0.02 (-0.05, 0.00) | -0.02 (-0.05, 0.01) |
|  | 17y | 0.00 (-0.04, 0.04) | -0.04 (-0.07, -0.01) | 0.02 (-0.01, 0.05) |
| Freq. Vegetables | 14y | 0.02 (-0.01, 0.05) | -0.02 (-0.04, 0.01) | -0.01 (-0.03, 0.02) |
|  | 17y | -0.01 (-0.05, 0.03) | -0.01 (-0.04, 0.03) | 0.00 (-0.04, 0.03) |
| Daily Fruit or Vegetable | 3y | -0.01 (-0.02, 0.00) | 0.00 (-0.01, 0.02) | 0.00 (-0.01, 0.01) |
| Portions Fruit per Day | 5y | 0.00 (-0.04, 0.03) | -0.01 (-0.03, 0.02) | -0.01 (-0.04, 0.02) |
|  | 7y | 0.02 (-0.01, 0.05) | 0.00 (-0.03, 0.02) | -0.01 (-0.04, 0.02) |
|  | 11y | 0.01 (-0.02, 0.05) | -0.04 (-0.07, -0.01) | -0.01 (-0.04, 0.02) |

**Table C: Descriptive statistics by sample.** ‘All’ refers to the full eligible sample (n = 15,180); ‘Genetic Trios’ to the sample with genotyped mother-father-offspring triads (n = 2,562); and ‘Mother-Offspring Pairs’ to the sample with genotyped mothers and offspring (n = 5,184). Descriptive statistics are weighted with recruitment weights accounting for the cluster stratified sampling design. Wald tests for each variable were carried out to assess whether there were mean level differences between ‘Genetic Trios’ and non-Genetic Trios and between Mother-Offspring Pairs and non-Mother-Offspring Pairs. *** p < 0.01; ** p < 0.01; * p < 0.01.

|  | | Eligible Sample | | Genetic Trios | | Mother-Offspring Pairs | |
| --- | --- | --- | --- | --- | --- | --- | --- |
|  | Variable | Mean (SD) / n (%) | Missing % | Mean (SD) / n (%) | Missing % | Mean (SD) / n (%) | Missing % |
|  | Birthweight (g) | 3424.9 (519) | 1.6% | 3436.1 (513.3)*** | 0.9% | 3424 (518.4)** | 1.1% |
|  | BMI (Z-Score) @ 3y | 0.5 (0.9) | 25.9% | 0.5 (0.9) | 12.9% | 0.5 (0.9) | 14.5% |
|  | BMI (Z-Score) @ 5y | 0.5 (0.9) | 21.8% | 0.4 (0.9) | 5% | 0.5 (0.9) | 6.3% |
|  | BMI (Z-Score) @ 7y | 0.4 (1) | 28.2% | 0.4 (1) | 4.8% | 0.4 (1) | 7.7% |
|  | BMI (Z-Score) @ 11y | 0.5 (1.2) | 32.8% | 0.5 (1.2) | 3.5% | 0.5 (1.2) | 5.8% |
|  | BMI (Z-Score) @ 14y | 0.5 (1.2) | 43.3% | 0.5 (1.2) | 3.1% | 0.6 (1.2) | 3.5% |
|  | BMI (Z-Score) @ 17y | 0.5 (1.2) | 52.3% | 0.5 (1.2) | 16.4% | 0.6 (1.3) | 22.9% |
|  | Body Fat (%) @ 7y | 20.6 (4.7) | 29.5% | 20.5 (4.7) | 6.2% | 20.6 (4.6)** | 9.3% |
|  | Body Fat (%) @ 11y | 21.7 (7.4) | 33.4% | 21.5 (7.4) | 4% | 21.8 (7.5)** | 6.6% |
|  | Body Fat (%) @ 14y | 21.2 (8.9) | 43.7% | 21.2 (8.8) | 3% | 21.4 (9)** | 3.7% |
|  | Body Fat (%) @ 17y | 21.6 (10) | 52.8% | 21.5 (9.5) | 16.9% | 21.7 (10.1)** | 23.6% |
|  | Fat-to-Non-Fat Ratio @ 7y | 0.3 (0.1) | 29.4% | 0.3 (0.1) | 6.2% | 0.3 (0.1)** | 9.3% |
|  | Fat-to-Non-Fat Ratio @ 11y | 0.3 (0.1) | 33.7% | 0.3 (0.1) | 4.5% | 0.3 (0.1)** | 7.1% |
|  | Fat-to-Non-Fat Ratio @ 14y | 0.3 (0.1) | 44.1% | 0.3 (0.1) | 3.7% | 0.3 (0.2)** | 4.5% |
|  | Fat-to-Non-Fat Ratio @ 17y | 0.3 (0.2) | 53.3% | 0.3 (0.2) | 17.5% | 0.3 (0.2)** | 24.5% |
|  | Waist-to-Height Ratio @ 5y | 0.5 (0) | 22.1% | 0.5 (0)** | 5% | 0.5 (0) | 6.5% |
|  | Waist-to-Height Ratio @ 7y | 0.5 (0) | 29% | 0.5 (0)** | 5.3% | 0.5 (0) | 8.4% |
|  | Diet Factor @ 11y | 0 (1) | 30.8% | 0 (1)*** | 1.6% | 0 (1) | 3.8% |
|  | Diet Factor @ 14y | 0 (1) | 41.4% | 0 (1)*** | 1.2% | -0.1 (1) | 1.2% |
|  | Diet Factor @ 17y | 0 (1) | 64.7% | 0 (1)*** | 35% | -0.1 (1) | 41.7% |
| Sex | Male | 7,948.3 (51.4%) | 0% | 1,347.3 (51.2%) | 0% | 2,764.9 (51.6%)* | 0% |
|  | Female | 7,506.7 (48.6%) |  | 1,282.7 (48.8%) |  | 2,592.1 (48.4%) |  |
|  | Gestation Age (Months) | 9.1 (0.4) | 3.7% | 9.1 (0.5)* | 2.1% | 9.1 (0.4)* | 3.2% |
|  | Maternal Age at Birth | 29.4 (5.9) | 0.5% | 29.9 (5.3)*** | 0.3% | 29.4 (5.8)*** | 0.4% |
|  | Mother's BMI | 24.8 (4.8) | 3.2% | 25 (4.7)** | 1.2% | 24.8 (4.8)*** | 1.4% |
|  | Father's BMI | 26.1 (3.9) | 21.9% | 26.1 (3.9)** | 1.8% | 26.2 (3.9)** | 14.9% |
| Family Social Class (NS-SEC) | Managerial and Professional | 6,731.4 (44.7%) | 2.6% | 1,250.3 (47.5%)*** | 0% | 2,400.3 (44.8%)*** | 0% |
|  | Intermediate | 1,428.0 (9.5%) |  | 277.7 (10.6%) |  | 518.3 (9.7%) |  |
|  | Small Employer and Self-Employed | 1,118.3 (7.4%) |  | 210.6 (8%) |  | 409.4 (7.6%) |  |
|  | Lower Supervisory & Technical | 1,261.1 (8.4%) |  | 234.3 (8.9%) |  | 448.8 (8.4%) |  |
|  | Semi-Routine and Routine | 1,885.0 (12.5%) |  | 328.4 (12.5%) |  | 665.2 (12.4%) |  |
|  | Not Working | 2,637.1 (17.5%) |  | 328.6 (12.5%) |  | 913.2 (17.1%) |  |
|  | Mother's Years of Education | 6.2 (1.7) | 0.7% | 6.3 (1.7)*** | 0.2% | 6.2 (1.7)*** | 0.4% |
|  | Father's Years of Education | 6.1 (1.8) | 20.6% | 6 (1.8)*** | 1.6% | 6.1 (1.8)*** | 14.1% |
|  | School Readiness @ Age 3y | 61.7 (28.9) | 24.7% | 62.3 (28.3)*** | 11% | 61.1 (28.6)*** | 13.2% |
|  | BAS Naming @ Age 5y | 109.4 (18.1) | 19.4% | 110.1 (16.2)*** | 2.6% | 109 (18)*** | 4.2% |
| Country of Birth | England | 12,439.7 (80.5%) | 0% | 2,091.4 (79.5%) | 0% | 4,315.9 (80.6%)* | 0% |
|  | Wales | 855.4 (5.5%) |  | 152.6 (5.8%) |  | 298.4 (5.6%) |  |
|  | Scotland | 1,573.5 (10.2%) |  | 272.3 (10.4%) |  | 535.6 (10%) |  |
|  | Northern Ireland | 586.3 (3.8%) |  | 113.7 (4.3%) |  | 207.1 (3.9%) |  |

**Table D: Description of individual diet variables.**

| Variable | Survey Question | Labels | Variables | 3y | 5y | 7y | 11y | 14y | 17y |
| --- | --- | --- | --- | --- | --- | --- | --- | --- | --- |
| Daily Fruit or Vegetable | I would now like to ask you about things that relate to people's standard of living. Do you have any of the following items?....Fresh fruit or vegetables once a day for [child] | Yes; No | bpstfv00 | x |  |  |  |  |  |
| Portions Fruit per Day | On a typical day, how many portions of fresh, frozen, tinned or dried fruit does [child] eat? | None; One; Two; Three or more | cpfrtp00, dpfrtp00, epfrtp00 |  | x | x | x |  |  |
| Freq. Sugary Drinks | Parent Report: How often, if at all, does [child] drink sweetened drinks e.g. cola, squash or Sunny Delight?  Child Report: How often, if at all, do you drink sugary drinks like regular cola or squash? | More than once a day; Once a day; 3-6 days a week; 1-2 days a week; Less often but at least once a month; Less than once a month; Never; Don't know; Refused | epswtd00, fcswtd00, gcswtd00 |  |  |  | x | x | x |
| Freq. Sweetened Drinks | Parent Report: How often, if at all, does [child] drink artificially sweetened drinks e.g. diet cola, sugar-free squash?  Child Report: How often, if at all, do you drink diet drinks or sugar free drinks like diet cola or sugar-free squash? | More than once a day; Once a day; 3-6 days a week; 1-2 days a week; Less often but at least once a month; Less than once a month; Never; Don't know; Refused | epaswd00, fcaswd00, gcaswd00 |  |  |  | x | x | x |
| Freq. Fast Food | How often, if at all, do you eat fast food such as McDonalds, Burger King, KFC or other fast food like that? | More than once a day; Once a day; 3-6 days a week; 1-2 days a week; Less often but at least once a month; Less than once a month; Never | fctkwy00, gctkwy00 |  |  |  |  | x | x |
| Freq. Fruit | How often do you eat at least 2 portions of fruit per day?  A portion of fruit could be a whole piece of fruit, like an apple or banana or 80g of fruit (like in a fruit salad) but does not include fruit juices. | Never; Some days, but not all days; Every day | fcfrut00, gcfrut00 |  |  |  |  | x | x |
| Freq. Vegetables | How often do you eat at least 2 portions of vegetables including salad, fresh, frozen or tinned vegetables per day?  A portion is 3 heaped tablespoons of cooked vegetables or beans /pulses or a handful of cherry tomatoes or a small bowl of salad. It does not include potatoes. | Never; Some days, but not all days; Every day | fcvegi00, gcvegi00 |  |  |  |  | x | x |

# References

Kolenikov, S. (2010). Resampling Variance Estimation for Complex Survey Data. *The Stata Journal: Promoting Communications on Statistics and Stata*, *10*(2), 165–199. https://doi.org/10.1177/1536867X1001000201

Rao, J. N. K., & Wu, C. F. J. (1993). Bootstrap inference for sample surveys. *Proceedings of the Section on Survey Research Methods*.

Rubin, D. B. (1987). *Multiple Imputation for Nonresponse in Surveys*. John Wiley & Sons, Ltd. https://doi.org/10.1002/9780470316696

Silverwood, R. J., Narayanan, M., Dodgeon, B., Katsoulis, M., & Ploubidis, G. B. (2024). *Handling missing data in the CLS cohort studies* (p. 117) [User guide]. UCL Centre for Longitudinal Studies. https://cls.ucl.ac.uk/wp-content/uploads/2020/04/Handling-Missing-Data-User-Guide-2024.pdf

Staatz, C. B., Kelly, Y., Lacey, R. E., & Hardy, R. (2021). Area-level and family-level socioeconomic position and body composition trajectories: Longitudinal analysis of the UK Millennium Cohort Study. *The Lancet Public Health*, *6*(8), e598–e607. https://doi.org/10.1016/S2468-2667(21)00134-1

Wang, Y., Moreno, L. A., Caballero, B., & Cole, T. J. (2006). Limitations of the Current World Health Organization Growth References for Children and Adolescents. *Food and Nutrition Bulletin*, *27*(4_suppl5), S175–S188. https://doi.org/10.1177/15648265060274S502

1. Strictly, not all of the parents were eligible for inclusion in the genotyped sample as some were not biological parents or residing with their child at Sweep 6 (age 14y). However, it is not possible to accurately identify all parents who would have been eligible for genotyping because this would rely on knowing whether they were resident with their child at Sweep 6 - information that could only be obtained if the family remained in the study. [↑](#footnote-ref-1)
